# Navigating the Challenges of Metallopharmaceutical Agents: Strategies and Predictive Modeling for Skin Cancer Therapy

**DOI:** 10.3390/pharmaceutics18020145

**Published:** 2026-01-23

**Authors:** Fernanda van Petten Vasconcelos Azevedo, Ana Lúcia Tasca Gois Ruiz, Diego Samuel Rodrigues, Douglas Hideki Nakahata, Raphael Enoque Ferraz de Paiva, Daniele Ribeiro de Araujo, Ana Carola de La Via, Wendel Andrade Alves, Michelle Barreto Requena, Cristina Kurachi, Mirian Denise Stringasci, José Dirceu Vollet-Filho, Wilton Rogério Lustri, Vanderlei Salvador Bagnato, Camilla Abbehausen, Pedro Paulo Corbi, Carmen Silvia Passos Lima

**Affiliations:** 1 School of Medical Sciences, University of Campinas (UNICAMP), Campinas 13083-894, SP, Brazil; fvpetten@yahoo.com; 2 School of Pharmaceutical Sciences, University of Campinas (UNICAMP), Campinas 13083-871, SP, Brazil; ana.ruiz@fcf.unicamp.br; 3 School of Technology, University of Campinas (UNICAMP), Limeira 13484-332, SP, Brazil; diego.rodrigues@ft.unicamp.br; 4 Donostia International Physics Center—DIPC, 20018 Donostia, Gipuzkoa, Spain; douglas.nakahata@gmail.com (D.H.N.); raphael.enoque@gmail.com (R.E.F.d.P.); 5 Chemistry Department, Faculty of Sciences, Universitat Autònoma de Barcelona, 08193 Cerdanyola del Vallès, Barcelona, Spain; 6 Department of Biophysics, Paulista Medical School, Federal University of São Paulo (UNIFESP), São Paulo 04023-062, SP, Brazil; daniele.ribeiro@unifesp.br; 7 Center for Natural and Human Sciences, Federal University of ABC (UFABC), Santo André 09210-580, SP, Brazil; ana.carola@ufabc.edu.br (A.C.d.L.V.); wendel.alves@ufabc.edu.br (W.A.A.); 8 Institute of Physics of São Carlos (IFSC), University of São Paulo (USP), São Carlos 13566-590, SP, Brazil; requenamichelle@gmail.com (M.B.R.); cristina@ifsc.usp.br (C.K.); mirianstringasci@gmail.com (M.D.S.); volletfilho@gmail.com (J.D.V.-F.); vander@ifsc.usp.br (V.S.B.); 9 Institute of Biosciences, University of Araraquara (UNIARA), Araraquara 14801-340, SP, Brazil; wrlustri@uniara.edu.br; 10 Institute of Chemistry, University of Campinas (UNICAMP), Campinas 13083-862, SP, Brazil; camilla@unicamp.br (C.A.); ppcorbi@unicamp.br (P.P.C.)

**Keywords:** skin cancer, metallopharmaceuticals, chemical enhancers, microneedles, nanocarriers, liposomes, micelles, topical treatment, systemic treatment, systemic mathematical modeling

## Abstract

Skin cancer (SC) is the most prevalent malignancy worldwide, with subtypes varying in aggressiveness: basal cell carcinoma tends to be locally invasive, squamous cell carcinoma has a higher metastatic risk, and melanoma remains the deadliest form. Current treatments such as surgery, radiotherapy, and systemic chemotherapy are associated with aesthetic and functional morbidity, recurrence, and/or systemic toxicity. Although targeted therapies and immunotherapies offer clinical benefits, their high cost and limited accessibility underscore the need for innovative, affordable alternatives. Metal-based compounds (metallopharmaceuticals) are promising anticancer agents due to their ability to induce oxidative stress, modulate redox pathways, and interact with DNA. However, clinical translation has been limited by poor aqueous solubility, rapid degradation, and low skin permeability. This review discusses the most recent preclinical findings on gold, silver, platinum, palladium, ruthenium, vanadium, and copper complexes, mainly in topical and systemic treatments of SC. Advances in chemical and physical enhancers, such as hydrogels and microneedles, and in drug delivery systems, including bacterial nanocellulose membranes and nanoparticles, as well as liposomes and micelles, for enhancing skin permeation and protecting the integrity of metal complexes are also discussed. Additionally, we examine the contribution of photodynamic therapy to SC treatment and the use of mathematical and computational modeling to simulate skin drug transport, predict biodistribution, and support rational nanocarrier design. Altogether, these strategies aim to bridge the gap between physicochemical innovation and clinical applicability, paving the way for more selective, stable, and cost-effective SC treatments.

## 1. Introduction

Cancer is one of the leading causes of illness worldwide and accounts for nearly 10 million deaths annually. It is a group of diseases characterized by the uncontrolled growth of abnormal cells, which can invade nearby tissues and spread to other parts of the body [1,2,3,4].

Among all cancer types, skin cancer (SC) is the most frequently diagnosed in the world, with over 1.5 million new cases reported each year [4]. In Brazil, SC represents approximately 30% of malignant tumors, and projections estimate over 704,000 new cases annually [1].

**Figure 1:**
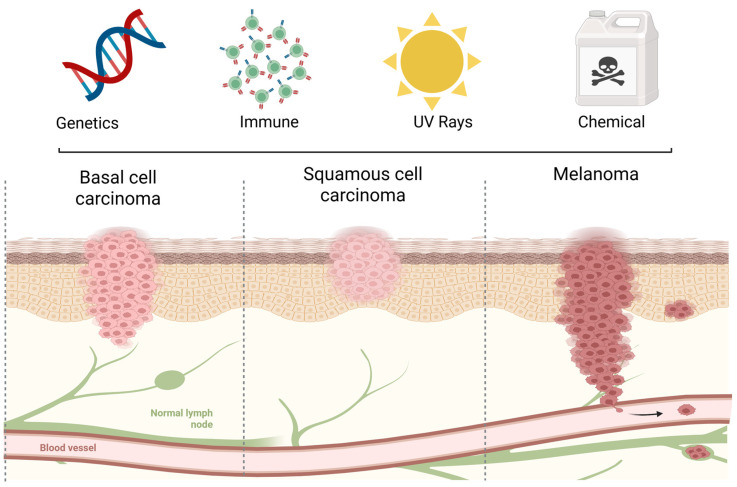
Schematic representation of the main etiological factors for skin cancer (genetic predisposition, immune dysfunction, ultraviolet radiation, and chemical exposure) and basal cell carcinoma, squamous cell carcinoma, and melanoma subtypes. Image created with BioRender. Van Petten, F. (2026). https://app.biorender.com/illustrations/67900e408505d292a0570b96 (accessed on 6 January 2026); inspired by Zeng (2023) [7].

Skin cancer incidence increases worldwide, particularly among older adults and fair-skinned individuals exposed to sunlight [5,6] and individuals from countries with a high socio-demographic index, such as those of North America, Northern Europe, and Oceania [3,5,6]. This kind of cancer is divided into two major types: non-melanoma (NM) and melanoma. Non-melanoma skin cancer (NMSC) includes basal cell carcinoma (BCC) and squamous cell carcinoma (SCC) (Figure 1).

### 1.1. Risk Factors for Skin Cancer

Several risk factors contribute to SC development and progression, which can be divided into two groups: biological and environmental factors (Figure 1).

#### 1.1.1. Biological Factors

Inherited conditions have been identified as important risk factors for SC, affecting several young individuals within the same family. Approximately 10% of patients inherit high-penetrance mutations and develop the hereditary forms of the tumors. Basal cell nevus syndrome or Gorlin–Goltz syndrome [8,9], characterized by mutations in the *PTCH1* gene, leads to multiple BCCs [10]. *Xeroderma pigmentosum* (XP), a rare disorder impairing DNA repair after ultraviolet (UV) exposure, strongly predisposes individuals to NMSC [11,12,13]. Hereditary melanoma is associated with mutations in the *MITF*, *CDKN2A*, *CDK4*, *MC1R*, *RB1*, *BRCA1*, or *BRCA2* genes [12,13,14,15]. It should also be considered that inherited low-penetrance mutations, mainly single-nucleotide variations (SNVs), that confer physical aspects, such as blue/green eyes, blond/red hair, and fair skin, predispose individuals to SC due to lower levels of melanin, which provide less natural protection against UV radiation [3]. SNVs can also determine abnormalities in metabolic processes, such as apoptosis, DNA repair, cell proliferation, cell pigmentation, angiogenesis, and immune system pathways, increasing predisposition to BCC [16,17], SCC [17,18] and melanoma [19,20,21,22,23,24].

Individuals who have undergone organ transplants, are receiving chronic immunosuppressive therapy (e.g., corticosteroids and cyclosporine), or are infected with the human immunodeficiency virus (HIV), become immunosuppressed and are at greater risk of developing SC or SC progression [24,25,26,27] when compared to individuals of the general population.

#### 1.1.2. Environmental Factors

The main environmental factor involved in skin carcinogenesis is chronic UV radiation exposure. Individuals with excessive sun exposure have a significant increase in risk of SC [25]. UVB radiation induces DNA damage by forming pyrimidine dimers and pyrimidinepyrimidone photoproducts, leading to mutations in key tumor suppressor genes, such as *TP53* [25]. UVA radiation induces indirect DNA damage by generating reactive oxygen species (ROS), thereby promoting the emergence of SC [26,27,28]. UV radiation can also indirectly affect the initiation, promotion, and progression of SC by inducing inflammation, in which the cyclooxygenase-2 (COX-2) protein plays a pivotal role [29,30,31].

Exposure to air pollutants, arsenic, selenium, zinc, and artificial lights has also been linked to increase of SC rates [32].

### 1.2. Types of Skin Cancer

#### 1.2.1. Non-Melanoma

Non-melanoma tumors arise from epidermal keratinocytes and account for approximately 99% of SCs [28,33].

**Figure 2:**
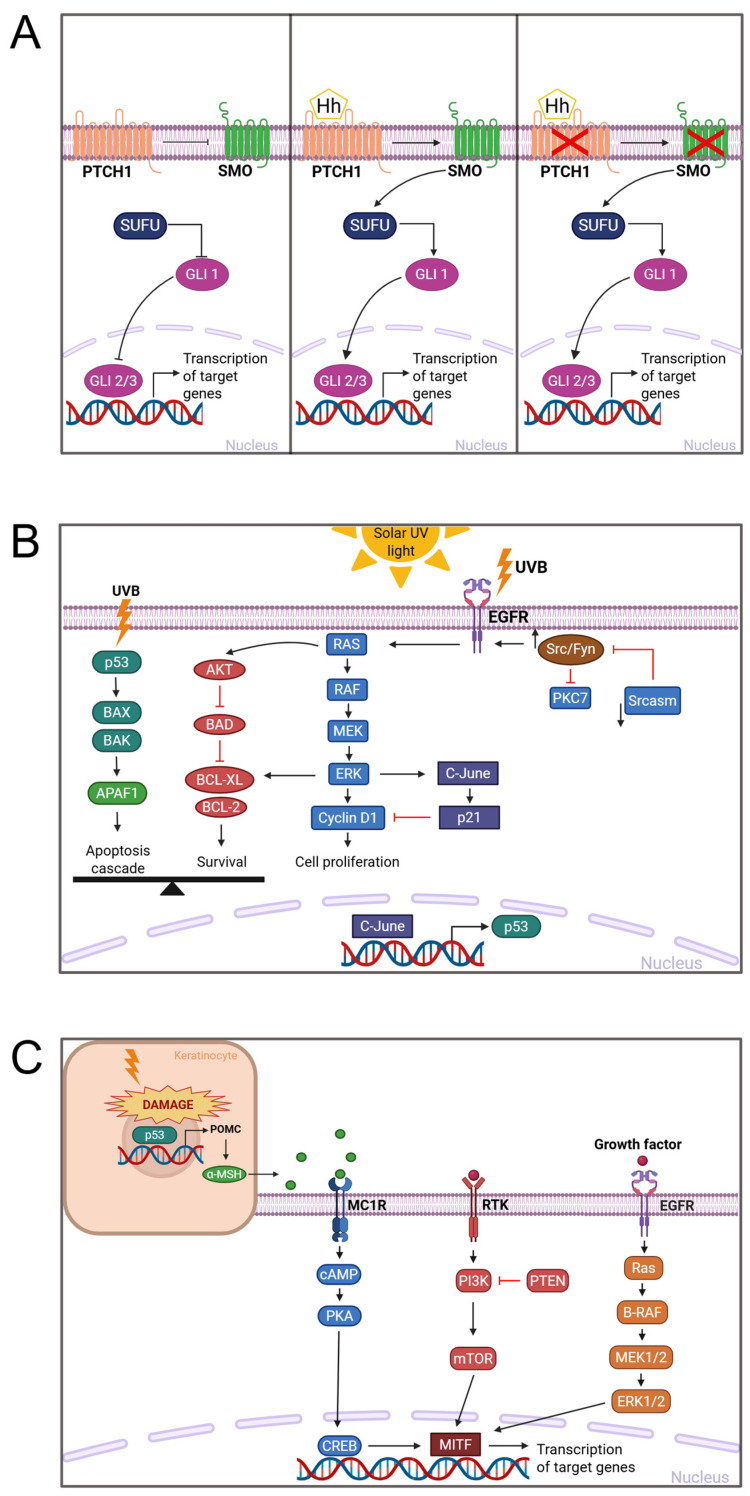
Molecular pathways involved in the pathogenesis of skin cancers. (**A**) The Hedgehog signaling pathway in basal cell carcinoma. In the absence of a Hedgehog ligand (Hh), PTCH1 inhibits SMO, allowing SUFU to retain GLI proteins in the cytoplasm and prevent the transcription of target genes. The binding of the ligand activates SMO, releasing GLI protein, which then translocates into the nucleus to activate transcription. Mutations in *PTCH1* or *SMO* activate the pathway and lead to uncontrolled gene expression, contributing to tumorigenesis. (**B**) Key molecular pathways in squamous cell carcinoma. Ultraviolet (UV) radiation induces mutations that stabilize and accumulate the p53 protein, triggering apoptotic pathways via BAX and APAF1 while also promoting survival through AKT and BCL2 signaling. Additional genetic alterations in *CDKN2A*, *NOTCH1/2*, *EGFR*, and *TERT* cooperate with aberrant RAS/RAF/MEK/ERK and PI3K/AKT/mTOR cascades, facilitating keratinocyte proliferation, survival, and tumor progression. (**C**) Melanoma signaling mechanisms. The image illustrates two interacting cell types: a keratinocyte (top left) and a melanocyte (bottom right), reflecting their crosstalk in response to UVB exposure. UVB-induced DNA damage activates p53 in keratinocytes, leading to the production of α-MSH, which binds to MC1R receptors on melanocytes. This activates the cAMP–PKA–CREB pathway and upregulates *MITF*, a master regulator of melanocyte survival and melanogenesis. Simultaneously, activating mutations in *BRAF* and *NRAS*, along with PI3K/mTOR and MAPK pathway activation, drive melanoma growth and progression. Image created in BioRender. Van Petten, F. (2026). https://app.biorender.com/illustrations/695d4fc40ea6a56294d93b7d (accessed on 6 January 2026); adapted from Khan (2022) [43].

Basocellular skin carcinoma arises in the basal layer of the epidermis and accounts for 80% of SCs [34,35]. Clinically, it often appears in the head and neck region, demonstrating local invasion and tissue destruction, but rarely metastasizes. Upregulation of the Hedgehog (HH) signaling pathway, driven mainly by mutations in PTCH1 or SMO [36,37], occurs in 90% of BCC cases and is central to tumor pathogenesis. A combined signature of *PTCH1*, *SMO*, and cytoplasmic release of GLI leads to increased cell proliferation and angiogenesis in BCC [15,38,39] (Figure 2A).

Squamous cell carcinoma arises from epidermal keratinocytes, accounts for ∼20% of SC, and is markedly more aggressive with a higher metastatic potential than BCC [40]. Mutations in *TP53*, primarily induced by UV exposure, are present in ≈95% of SCC cases leading to loss or dysfunction of the tumor suppressor protein p53 [11,41]. This dysfunction impairs the activation of pro-apoptotic proteins such as *BAX* and *APAF1,* favoring cell survival. A complex network involving p53, p16, *NOTCH1/NOTCH2*, *EGFR*, and *TERT,* along with pathways such as *RAS/RAF/MEK/ERK* and *PI3K/AKT/mTOR*, underpins SCC development [42,43] (Figure 2B).

#### 1.2.2. Melanoma

Melanoma originates from melanocytes, which are innate protectors against UV-induced damage. Though it comprises only 1% of SCs, it is the most aggressive form, with significant mortality rates in advanced disease [44,45,46,47]. Hallmarks include autonomous growth factor signaling, apoptosis resistance, limitless replication, angiogenesis, invasion, and metastasis [48]. These are driven by oncogene activation or tumor suppressor loss through mutations, deletions, translocations, epigenetic changes, and promoter methylation [48]. UV-induced DNA damage in keratinocytes upregulates *TP53* and stimulates α-MSH production, which then acts on neighboring melanocytes via MC1R, increasing cAMP and activating *CREB*, therefore inducing *MITF* via *CRTC* [49,50,51,52] Additionally, *BRAF* and *NRAS* mutations are key drivers in tumor development [53,54] (Figure 2C).

### 1.3. Treatment of Skin Cancer

#### 1.3.1. Modalities of Treatment

The treatment of small, localized SC is based on surgical resection; radiotherapy can replace surgery in specific cases. Cryoablation, which uses cold to destroy cancerous tissue, and photodynamic therapy (PDT), which uses light-activated drugs to target cancerous cells, are also considered for the treatment of patients with SC [55,56,57,58]. Despite their effectiveness, cryoablation and PDT have limitations in treatment depth, and PDT can cause skin irritation and prolonged light sensitivity [56].

A growing interest in the topical treatment of SC has been observed over the last decade with the purpose of avoiding mutilations imposed by surgery and toxic effects of non-surgical treatments [55,56,57]. 5-Fluorouracil (5-FU), a chemotherapeutic agent that blocks cell proliferation [56,59,60,61], and imiquimod, an immune response modifier that activates killer cells, macrophages, B-lymphocytes, and Langerhans cells, induce good responses in BCC and SCC but may be associated with skin irritation. Imiquimod is also more expensive than other agents [61,62]. Topical treatment of patients with melanoma has been viewed with reservation by most clinicians due to the metastatic potential of the tumor. Nevertheless, 5-FU/etoposide co-loaded with skin permeation enhancers and imiquimod have been seen as promising agents [62,63,64,65,66].

Patients with advanced SC receive systemic treatment. Cisplatin-based chemotherapy is the most used treatment for BCC and SCC; it offers modest clinical benefits and potentially severe toxicity [67,68]. Hedgehog signaling inhibitors (vismodegib and sonidegib) demonstrate good responses and low BCC recurrence [69,70,71]. Epidermal growth factor receptor (EGFR) inhibitors, including monoclonal antibodies such as cetuximab and panitumumab [67,72], and tyrosine kinase inhibitors (erlotinib, gefitinib, and dasatinib) [73] show clinical benefit and manageable toxicity in SCC in clinical trials. Cemiplimab, an anti-PD-1 systemic agent, emerges as an efficacious and tolerable option for BCC and SCC in phase I and II trials [42,67,71,73,74]. Inhibitors of *BRAF*, *MEK*, *c-KIT*, and *NRAS* show promise in melanoma. However, concerns about resistance and cutaneous toxicities highlight the need for close monitoring [75,76]. Immunotherapy with anti-PD1 and anti-CTLA4 agents (pembrolizumab, nivolumab, and ipilimumab) promotes the destruction of melanoma by cytotoxic T lymphocytes, with reduced recurrence and/or mortality in metastatic melanoma [44,46,47]. Still, resistance to immune checkpoint blockade is common [46,77].

#### 1.3.2. Limitations

Although agents with efficacy for SC are available on the market, the composition of the skin and cells and the characteristics of tumor carriers constitute unequivocal barriers to treatments [43].

**Figure 3:**
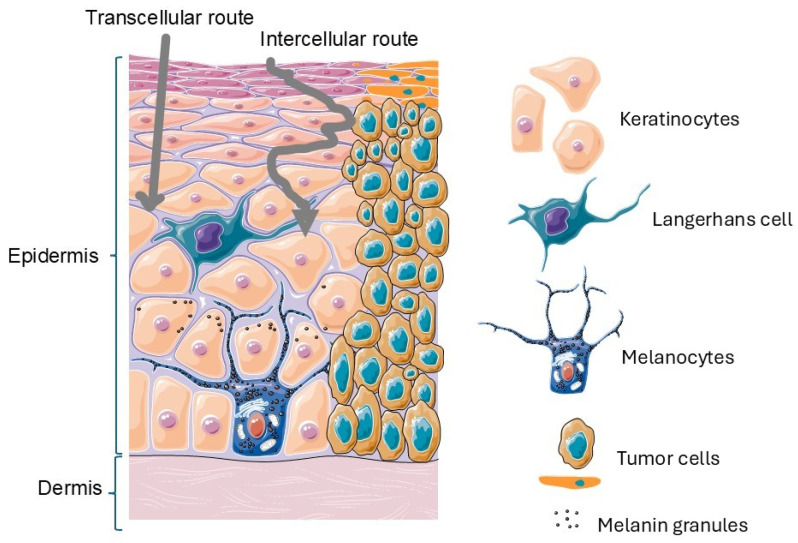
Schematic representation of the skin and a skin tumor. The illustration depicts keratinocytes, Langerhans cells, tumor cells, melanocytes, and melanin granules and highlights the transcellular and intercellular permeation routes. Adapted from Servier Medical Art (CC BY 4.0); created by the authors based on Alkilani (2015) [83].

One of the main challenges in the topical treatment of SC is the permeation of active compounds through the skin’s natural barriers. The anatomy of skin reveals that the uppermost layer of skin, the stratum corneum, is the main barrier that prevents the entry of anticancer agents into the skin; fatty cells, triglycerides, cholesterols, and ceramides dispose into a network that makes it difficult for the movement of molecules across the skin and hinder the action of topical treatments, and deep epidermal layers provide an extra skin protective barrier, with Langerhans cells showing immune response to agents used in the treatment of SC [78]. The cell membrane provides a biological barrier to anticancer drugs through phagocytosis and endocytosis [79]. The efficacy of an agent used in topical administration depends on its ability to overcome these barriers using the transcellular (intracellular), paracellular (intercellular), or transappendageal (skin appendages) routes [80,81] (Figure 3).

Cells of SC may also develop multidrug resistance (MDR) due to drug degradation, changes in drug receptors, DNA repair, and efflux pumps, resulting in a lack of response to treatment. The liver and kidneys provide another barrier to drug delivery to the tumor by removing therapeutic agents from the circulation [82].

Finally, an important point to consider in the topical and systemic treatments of SC is the high cost of most agents, which makes their use difficult for patients in general, particularly for those treated in public institutions.

Given the lack of efficient, safe, and readily available treatments for many SC patients worldwide, the search for alternative therapeutic agents and new procedures is mandatory. This review aims to highlight both the opportunities and limitations to define the future of metal-based therapeutics, the advances in chemical and physical enhancers, and drug delivery systems for enhancing skin permeation and protecting the integrity of metal complexes, as well as examine the contribution of photodynamic therapy in the treatment of SC and mathematical and computational modeling to simulate skin drug transport.

## 2. Metallopharmaceuticals in Skin Cancer

Metal-based drugs, also known as metallopharmaceuticals, have gained considerable attention in recent years as a promising approach for the treatment of patients with SC. These compounds exploit the unique biochemical properties of metal centers to induce cytotoxicity in tumor cells. Metal-based agents exert their anticancer effects through multiple mechanisms, including induction of apoptosis, inhibition of thioredoxin reductase, disruption of mitochondrial function, and generation of ROS [84,85,86,87,88,89]. Additionally, many metal complexes directly damage DNA by forming adducts or inducing strand breaks, further impairing tumor cell survival [27]. The diverse modes of action of these compounds make them attractive candidates for overcoming drug resistance and improving therapeutic outcomes in SC treatment.

The development of metallopharmaceuticals for the treatment of patients with SC must navigate multiple hurdles, including poor solubility, low bioavailability, and off-target toxicities. To overcome these limitations, advanced formulation strategies, such as the use of topical application vehicles, microneedles (MNs), and bacterial nanocellulose membranes (BNCMs), have been proposed to enhance their pharmacokinetics and biodistribution. Notably, PDT offers significant opportunities to improve efficacy in localized treatments and has also been considered for SC patients. In vivo studies play a critical role in validating the therapeutic potential of metallopharmaceuticals, allowing researchers to explore their interactions within the tumor microenvironment and the broader systemic effects [90,91].

In this context, we provide an overview of the latest advances in the formulation and delivery of metallopharmaceuticals for SC, focusing particularly on in vivo findings that are crucial to shaping their translational potential. The discussion covers the mechanisms of action of metal-based compounds, new approaches to topical delivery, challenges associated with topical and systemic delivery, and the integration of nanotechnology to optimize drug performance. In addition, the contribution of PDT as a treatment for SC and of mathematical and computational modeling to simulate skin drug transport, predict biodistribution, and support rational nanocarrier design are also covered in this review.

By addressing these key aspects, this review aims to highlight both the opportunities and limitations to define the future of metal-based therapeutics in SC.

### 2.1. Metallopharmaceuticals for Topical Treatment

New metal-based compounds have been synthesized for the topical treatment of SC. In this review, a series of platinum, palladium, gold, copper, rhodium, and ruthenium complexes was identified as possible alternatives for treating SC patients, opening a broad field of investigation in this area. Across preclinical studies, topical metallodrug strategies achieved meaningful local control while minimizing systemic exposure. In particular, BNCMs carrying silver(I)–nimesulide stabilized the complex, improved photostability, and enabled sustained cutaneous release with no added local irritation [92]; MN-assisted and hydrogel/film vehicles increased epidermal/dermal deposition; and photo-triggerable Ru/Pt platforms provided spatially confined cytotoxicity in superficial lesions, together outlining a credible path for localized, patient-sparing therapy [87,93,94,95,96].

During formulation development of new metallodrugs (definitions used in this review: “topical” denotes local delivery without measurable plasma exposure; “transdermal” denotes delivery via skin into systemic circulation; and “systemic” includes oral/IV administration), an important point to be taken into consideration is their physicochemical characteristics, such as their hydrophilicity, solubility, and oil/water partition coefficient. Moreover, the physiopathological conditions of the skin, such as hydration, age, and lipid film, also modulate the absorption of a substance. These parameters will assist in the selection of the pharmaceutical form and excipients that will assist in selecting the pharmaceutical form and excipients that will make up innovative formulations enabling both sustained release and controlled permeation of metallodrugs [81,97].

A favorable therapeutic index is central for the clinical relevance of topical skin cancer therapies because efficacy must be balanced against collateral damage to surrounding normal skin. In standard topical regimens used in non-melanoma skin cancer and field cancerization settings, local skin reactions such as erythema, pain or stinging/burning, crusting/scabbing, and erosions/ulcerations are common and can be treatment limiting, directly affecting tolerability and adherence [98,99,100]. Accordingly, emerging topical metallopharmaceutical platforms should be discussed not only in terms of delivery and local efficacy but also through standardized reporting of local tolerability and off-target effects in adjacent healthy tissue. When strategies specifically designed to increase penetration are employed, it is prudent to confirm that clinically meaningful systemic exposure does not occur [100].

Beyond determinants of exposure related to formulation and skin physiology, it is also important to recognize that “skin cancer” comprises biologically distinct entities. In the current topical metallopharmaceutical literature, non-melanoma skin cancers, particularly SCC and BCC, predominate as experimental models, consistent with the global burden of non-melanoma skin cancers (NMSCs) [101]. This emphasis also reflects the practical suitability of these lesions for skin-directed platforms, as they are often clinically accessible and amenable to local delivery strategies, for example, membranes or other topical devices [92]. By contrast, melanoma remains comparatively under-represented in topical metallodrug studies, and this may partly reflect that the management of advanced melanoma is largely systemic and increasingly guided by clinically relevant features and biomarkers, such as BRAF status, PD-L1 expression, brain metastases, and performance status [44]. Collectively, these considerations reinforce that future progress in topical metallopharmaceuticals should integrate delivery optimization with subtype-specific biology and, where feasible, biomarker-informed stratification to support translational study design and outcome interpretation [42].

### 2.2. Metallopharmaceuticals for Systemic Treatment

**Figure 4:**
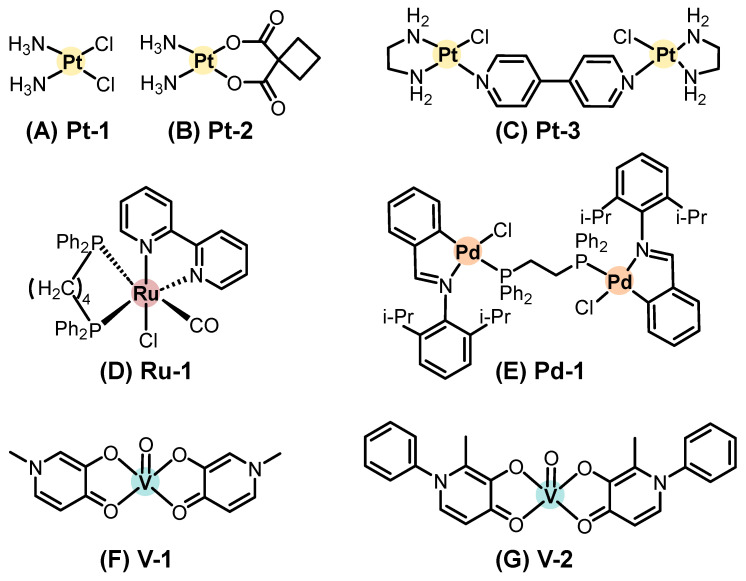
Structural representations of metal-based compounds evaluated for systemic treatment of skin cancer. (**A**) Cisplatin (Pt-1), a platinum(II) complex used clinically for cutaneous squamous cell carcinoma (SCC), inducing DNA crosslinking and apoptosis. (**B**) Carboplatin (Pt-2), a platinum(II) complex with improved pharmacokinetics and reduced nephrotoxicity, under investigation for combination regimens in melanoma and SCC. (**C**) [{Pt(en)Cl}_2_(μ-4,4′-bipy)]Cl_2_·2H_2_O (Pt-3), a dinuclear platinum(II) complex showing potent antimetastatic and antiangiogenic activities in melanoma xenografts. (**D**) [RuCl(CO)(dppb)(bipy)]PF_6_ (Ru-1), a ruthenium(II) carbonyl complex with selective cytotoxicity against melanoma cells and in vivo tumor volume reduction. (**E**) [{ClPd(C_6_H_4_)CH = N(2,6-di-iPr-C_6_H_3_)}_2_(μ-Ph_2_P(CH_2_)_2_PPh_2_)] (Pd-1), a binuclear cyclopalladated complex that induces apoptosis and autophagy through MAPK pathway activation. (**F**) Vanadium-based compounds: V-1 ([VO(mpp)_2_]) and (**G**) Vanadium V-2 ([VO(ppp)_2_]), which demonstrate enhanced cytotoxicity against human melanoma cell lines. Structures were drawn using ChemDraw (online version 22.2.0).

**Figure 5:**
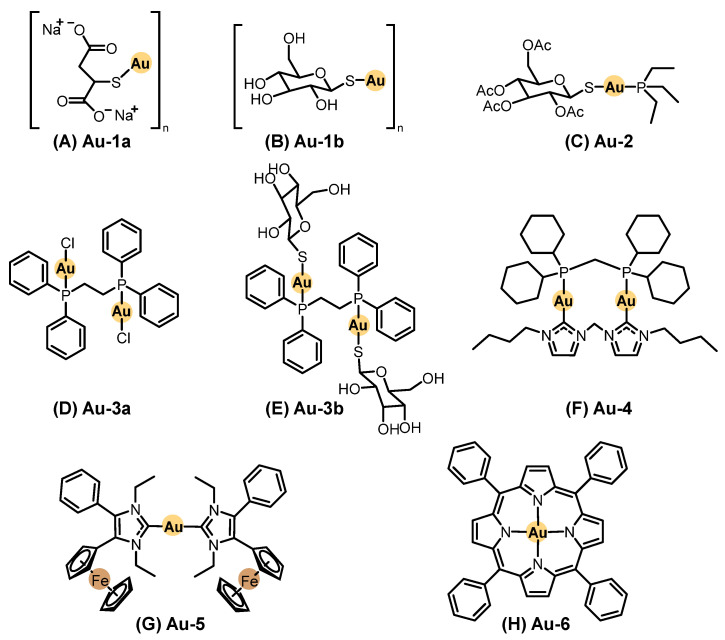
Structural representations of gold(I) and gold(III) complexes evaluated in vivo in melanoma-bearing mouse models. (**A**) Au-1a and (**B**) Au-1b: polymeric and sugar-based gold(I) thiolates with anti-inflammatory origin. (**C**) Au-2 (auranofin): a clinical gold(I) complex with phosphine–thioglucose ligands. (**D**) Au-3a and (**E**) Au-3b: bisphosphine gold(I) complexes with or without sugar-functionalized ligands. (**F**) Au-4: dinuclear gold(I) NHC–diphosphane cationic complex. (**G**) Au-5: biscarbene–ferrocenyl gold(I) complex designed for increased lipophilicity. (**H**) Au-6: porphyrin-based gold(III) complex with square planar geometry. Structures were drawn using ChemDraw (online version 22.2.0).

**Figure 6:**
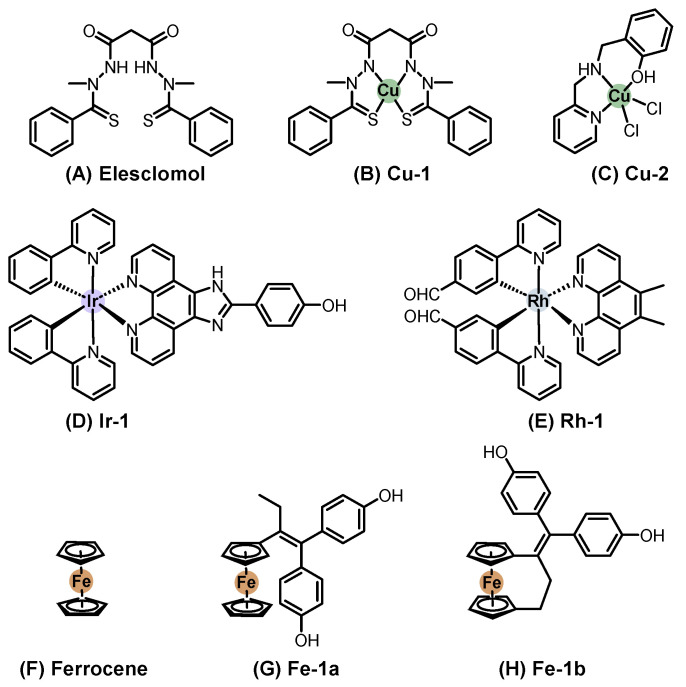
Structures of metal-based complexes evaluated in vivo for melanoma treatment. (**A**) Elesclomol (ligand); (**B**) Cu-1 (copper–elesclomol complex); (**C**) Cu-2 (phenol–pyridine chelate); (**D**) Ir-1 (iridium(III) complex with polypyridyl ligands); (**E**) Rh-1 (rhodium(III) complex); (**F**) ferrocene (Fe); (**G**,**H**) ferrocene–tamoxifen conjugates (Fe-1a and Fe-1b, ferrocifens). Structures were drawn using ChemDraw (online version 22.2.0).

Systemic chemotherapy involves administering agents orally or intravenously to distribute the drug throughout the body. This treatment modality has been extensively applied across various types of cancer, including SC. In contrast to topical approaches, a wider variety of metal-based compounds have been investigated under preclinical and clinical settings for systemic treatment of SC. Representative chemical structures of metal-based compounds evaluated in systemic chemotherapy for SC with demonstrated in vitro and/or in vivo antitumor activity, including platinum, ruthenium, palladium, vanadium, gold, copper, and other metal-based compounds, are presented in Figure 4, Figure 5 and Figure 6. Below, we highlight some of the most notable examples bringing in vivo data and evaluated in systemic chemotherapy.

#### 2.2.1. Platinum-Based Compounds

Cisplatin (*cis*-[PtCl_2_(NH_3_)_2_], Pt-1, Figure 4A), a platinum(II) complex introduced into oncology practice in 1978, remains a landmark drug in cancer treatment, including for locally advanced, recurrent, or metastatic cutaneous SCC [102].

Another clinical platinum(II) complex, carboplatin ([Pt(CBDCA)(NH_3_)_2_], where CBDCA is cyclobutene dicarboxylate, Pt-2, Figure 4B), has been evaluated primarily in gastrointestinal malignancies. Still, preclinical studies have suggested that its favorable pharmacokinetics and reduced nephrotoxicity may support future exploration in combination regimens for SC [87].

More recently, Adhikari (2024) [103] reported the synthesis and evaluation of a dinuclear platinum(II) complex, [{Pt(en)Cl}_2_(μ-4,4′-bipy)]Cl_2_·2H_2_O (Pt-3, Figure 4C), where en is ethylenediamine and bipy is bipyridine. This complex exhibited potent in vitro cytotoxicity and was able to inhibit the growth of melanoma xenografts in zebrafish models, notably blocking tumor neovascularization and metastasis [103,104]. Although this study was not conducted in SCC models, its antiangiogenic and antimetastatic performances reinforce the relevance of platinum-based metallodrugs in systemic anticancer strategies and may inform future applications in SCC therapy.

#### 2.2.2. Ruthenium-Based Compounds

A novel ruthenium(II) complex, [RuCl(CO)(dppb)(bipy)]PF_6_ (Ru-1, Figure 4D), was synthesized by Carnizello (2016) [105]; in this compound, dppb denotes 1,4-bis(diphenylphosphino)butane. Ru-1 displayed potent antiproliferative activity against several human cancer cell lines, including cervical adenocarcinoma (HeLa), breast adenocarcinoma (MCF7), glioblastoma (MO59J), hepatocellular carcinoma (HepG2), and murine melanoma (B16F10), while showing selectivity toward melanoma cells compared with non-tumoral V79 fibroblasts. In vivo, the treatment of C57BL/6 mice bearing B16F10 tumors with Ru-1 (5.0 mg/kg, daily for five days) reduced tumor volume by approximately 60% and markedly decreased mitotic figures in histological analyses compared with controls [105].

#### 2.2.3. Palladium-Based Compounds

Aliwaini (2013) [106] described the biological evaluation of a binuclear cyclopalladated complex, [{ClPd(C_6_H_4_)CH = N(2,6-di-iPr-C_6_H_3_)}_2_(μ-Ph_2_P(CH_2_)_2_PPh_2_)] (Pd-1, Figure 4E), in the inhibition of the proliferation of melanoma cell lines ME1402, WM1158, and 501-mel, achieving IC_50_ values between 0.19 and 0.25 μmol·L^−1^, and exhibited selectivity compared to fibroblasts.

In vivo studies were conducted by subcutaneously injecting ME1402 melanoma cells into six-week-old nude mice. After tumor establishment, Pd-1 and cisplatin were administered intraperitoneally twice a week for two weeks. Treatment with Pd-1 led to a 90% reduction in tumor size and 85% reduction in tumor weight, outperforming cisplatin, which achieved reductions of 56.4% and 63.6%, respectively [106].

Mechanistic investigations suggested that Pd-1 induces both apoptosis and autophagy through activation of the P38 and ERK MAPK pathways [106].

#### 2.2.4. Vanadium-Based Compounds

Vanadium-based compounds have been evaluated as therapeutic candidates for melanoma treatment. Vanadyl sulfate (VOSO_4_) and vanadium pentoxide (V_2_O_5_) exhibited antiproliferative activity against B16F10 murine melanoma cells, either alone or in combination with the recombinant Newcastle disease virus (NDV), an oncolytic virus that selectively targets tumor cells while sparing healthy tissue [107,108].

Additionally, two vanadium(IV) complexes, [VO(mpp)_2_] (V-1, Figure 4F) and [VO(ppp)_2_] (V-2, Figure 4G), where mpp is 1-methyl-3-hydroxy-4(1H)-pyridinone and ppp is 1-phenyl-2-methyl-3-hydroxy-4(1H)-pyridinone, demonstrated in vitro antiproliferative activity against A375 and CN-mel human melanoma cell lines. These findings highlight the importance of ligand design in optimizing the antitumor potential of vanadium compounds [109,110].

In vivo experiments conducted in C57BL/6 mice implanted intradermally with melanoma cells showed promising results. Treatment with vanadyl sulfate (40 mg/kg) combined with NDV led to tumor regression within 96 h. Furthermore, intraperitoneal administration of V_2_O_5_ (10 mg/kg, once weekly) prolonged survival in tumor-bearing mice without inducing organ toxicity, supporting its systemic safety profile [107,108].

#### 2.2.5. Gold-Based Compounds

Gold complexes have been extensively studied as anticancer agents, particularly following the clinical use of gold thiolates (Au-1a and Au-1b, Figure 5A,B) and auranofin (Au-2, Figure 5C) for the treatment of rheumatoid arthritis. Although originally developed for inflammatory diseases, these agents inspired investigations into gold-based anticancer pharmacophores, as summarized by Manzano (2022) [87]. Still, no clinical trials evaluating gold-based agents on SC have been reported to date.

For this article, we considered the gold(I) and gold(III) compounds evaluated in melanoma-bearing animal models, including both clinically tested and experimental scaffolds. To understand their biological performance, it is important to review the coordination chemistry of gold. Gold primarily exists in +1 and +3 oxidation states. Gold(I) complexes exhibit a linear coordination geometry and bind to soft donor atoms such as those from phosphines, carbenes, and halides. In contrast, gold(III) forms square planar complexes stabilized by chelating ligands with intermediate hardness [87,111].

Among gold compounds, Au-2 is the most extensively investigated molecule. Although widely studied in vitro in SC models, the in vivo performance remains modest. Mirabelli et al. [112] evaluated auranofin in murine B16-F10 melanoma models but found no significant extension of survival after intraperitoneal or subcutaneous administration. Conversely, Stafford et al. [113] reported that auranofin reduced tumor volume by 60% in a FaDu xenograft Balb/C model after four days of treatment with 10 mg/kg intraperitoneally, twice daily. Following these studies, bisphosphine gold(I) compounds (Au-3a and Au-3b, Figure 5D,E) of the general structure Au_2_(dppe)X_2_ were developed. These were tested in B16 melanoma models by intraperitoneal (i.p.) injection (10 mg/kg/day), resulting in a 30% increase in lifespan. Another derivative, [Au(dppe)_2_]Cl, extended survival by 50% when administered at 1.9 µmol/kg/day for ten days [112,113].

N-heterocyclic carbene (NHC) gold(I) complexes, such as Au-4 (Figure 5F), a dinuclear NHC–diphosphane cationic complex, reduced tumor volume by 62% in C57BL/6N mice bearing B16-F10 melanoma after eight days of treatment (15 mg/kg i.p., every two days) [114]. Biscarbene complexes, such as Au-5 (Figure 5G), incorporating ferrocenyl groups to enhance lipophilicity, reduced tumor volume by 18–48% in Balb/C xenografts after four i.p. doses over 12 days [115].

In the area of gold(III) chemistry, a porphyrin–gold(III) complex (Au-6, Figure 5H) demonstrated significant tumor suppression in C57BL/6 mice implanted with B16-F10 melanoma. Treatment with 0.125–0.25 mg/kg/day over ten days led to tumor sizes three times smaller than untreated controls [114].

Overall, while gold-based compounds show potential against SC, the translation of in vitro potency into in vivo efficacy remains a challenge. However, further studies addressing pharmacokinetics, biodistribution, and toxicity of gold-based compounds are crucial before clinical development [116].

#### 2.2.6. Copper-Based Compounds

Copper(I) complexes can adopt structural features like those of Au(I), including linear NHC complexes and phosphane-based ligands [117]. However, the chemistry of copper(I) differs significantly from that of gold(I). Copper(I) preferentially forms tetrahedral geometries and has a strong tendency to oxidize to copper(II), whereas gold(I) is more resistant to redox changes in biological environments. Several copper(I) complexes have been investigated in vitro for SC treatment, though no in vivo data have been reported. It is important to note that copper(I) compounds are often toxic, leading to promising low IC_50_ values in vitro but limited applicability in vivo [118].

On the other hand, copper(II) complexes form more stable structures when coordinated with chelating agents [119]. The pronounced Jahn–Teller effect further stabilizes these compounds. Among the many copper(II) complexes tested in vitro against SC cells, one of the most significant in vivo studies involved the ligand elesclomol (Figure 6A) with copper(II), resulting in Cu-1 (Figure 6B). Elesclomol is a small organic molecule that advanced to clinical trials for melanoma chemotherapy. A phase III clinical trial was conducted in patients with stage IV melanoma and high lactate dehydrogenase (LDH) levels (*n* = 651). It was compared to a combination of elesclomol with paclitaxel versus paclitaxel alone. While the study concluded that elesclomol did not improve progression-free survival, it confirmed that elesclomol correlates with LDH baseline levels, establishing it as a predictive biomarker [120].

Mechanistic studies later revealed that elesclomol binds strongly to copper in the serum, transporting it to the mitochondria, where copper(II) is reduced to copper(I), generating ROS and triggering apoptosis [121]. Subsequently, copper(II)–, nickel(II)–, and platinum(II)–elesclomol complexes were synthesized and evaluated in vitro. The copper(II) complex was found to be 34-fold and 1040-fold more potent than the nickel(II) and platinum(II) complexes, respectively. Additionally, Cu-1 was shown to oxidize ascorbic acid under physiological conditions, further supporting its redox activity [122].

More recently, Liu et al. [123] developed ^64^Cu(II) elesclomol as a theranostic agent for hypoxic tumors. It was tested in prostate carcinoma (22Rv1) and glioblastoma (U84-MG) xenografts in BALB/c nude mice. In the prostate cancer model, the compound successfully inhibited tumor growth. Although these results are not related to SC, they demonstrate the potential of Cu-1 as a theranostic agent, suggesting an avenue for future research in melanoma therapy [123].

Other copper(II) complexes incorporating common chelating ligands, such as 1,10-phenanthroline (phen), bispyridine-2-methylamine, and pyridine-2-methylamino-phenol, have also been evaluated. Among them, Cu-2 (Figure 6C) was tested in vivo in C57BL/6 mice injected subcutaneously with B16-F10 melanoma cells. Before treatment, its LD_50_ was determined at 179.4 mg/kg. The therapeutic protocol involved intraperitoneal (i.p.) injections of 45 mg/kg every day for 21 days. Cisplatin, used as positive control, was administered at 0.5 mg/kg under the same conditions. The Cu-2 complex achieved an 87% reduction in tumor volume compared to 80% with cisplatin, showing strong antitumor efficacy [124].

#### 2.2.7. Other Metal-Based Compounds Derivatives

Iridium and rhodium, both from the same transition metal family, share similarities but also exhibit distinct differences in their chemistry. In their +3 oxidation state, they are highly stable. Iridium(III) complexes typically adopt octahedral or piano stool geometries (when coordinated with an arene ligand). They are widely explored as photosensitizers for PDT due to their high triplet-state quantum yields [56,125].

Iridium polypyridyl complexes are a promising class for both chemotherapy and PDT applications. The compound Ir-1 (Figure 6D) and its liposomal formulation (Ir-1-lipo) were investigated in vivo in a C57BL/6 mouse model bearing B16 melanoma tumors. Ir-1-Lipo showed a tumor inhibitory rate of 72.55% compared with the untreated control group, supporting liposomal delivery as a viable strategy to enhance the therapeutic potential of Ir(III) complexes in melanoma [125].

Rhodium, Rh-1 (Figure 6E), was tested in Balb/C nude mice implanted with A375 melanoma xenografts. Once palpable tumors developed, the mice received subcutaneous injections of Rh-1 (75 mg/kg) three times per week for 35 days. At the end of the study, tumor volume was reduced by 60% compared to the control group, with no systemic toxicity [126].

Ferrocene-based compounds have been widely explored in medicinal chemistry. Despite containing iron(II), ferrocene (Figure 6F) exhibits chemical behavior more like that of aromatic organic compounds than conventional iron complexes. Its high lipophilicity, via redox activity, makes it an attractive scaffold for anticancer drug design [127].

Ferrocene was tested in a C57BL/6 mouse model of lung metastases induced by B16 melanoma cells. The mice received intraperitoneal injections of ferrocene at three different doses (0.5, 1.0, and 2.0 mg/kg) on days 1, 8, and 15. Lung volume and weight were measured after 25 days. The results showed a 58% reduction in total lung weight, closely resembling the lungs of tumor-free mice [127].

Ferrocene–tamoxifen conjugates (Fe-1a and Fe-1b, Figure 6G and Figure 6H, respectively), known as ferrocifens, have also been investigated as potential anticancer agents. Resnier (2017) formulated the ferrocene complexes Fe-1a and Fe-1b in lipid nanocapsules (LNCs) and co-encapsulated them with Bcl-2 siRNA to modulate anti-apoptotic protein expression [128]. These formulations were evaluated in NMRI mice bearing SK-Mel28 melanoma xenografts, established by subcutaneous injection of cancer cells that were allowed to develop for three weeks. Groups of mice received intravenous injections of the formulations for five consecutive days, with two-day intervals, repeated for three weeks. The formulations were administered at 45 mg/kg, while dacarbazine (DTIC) served as a positive control at 100 mg/kg. It was observed that Fe-1b and DTIC each reduced tumor weight by 30%, whereas siRNA alone reduced tumor weight by 20%. However, the combination of Fe-1b and siRNA resulted in a 50% reduction in tumor weight, suggesting a synergistic effect between the agents [128].

The evidence highlights the versatility of non-platinum metals in SC therapy and encourages further research to discover their mechanisms of action and delivery platforms.

#### 2.2.8. Comparative Insights

Overall, the systemic use of metallodrugs in skin cancer (SC), particularly melanoma and cutaneous SCC, highlights both the chemical diversity and mechanistic versatility of metal-based chemotherapeutics. Platinum-based compounds remain the clinical benchmark, primarily acting through covalent DNA binding and apoptosis induction via DNA crosslinking and damage recognition pathways. However, newer platinum(II) and platinum(IV) architectures, including multinuclear and ligand-modified derivatives, aim to enhance selectivity and overcome resistance. Ruthenium, vanadium, gold, and copper complexes, on the other hand, exert strong antiproliferative effects by modulating thiol-dependent enzymes, redox balance, and mitochondrial respiration, frequently accompanied by reactive oxygen species (ROS) generation.

From a comparative standpoint, each metal center confers distinct pharmacological and mechanistic traits, shaping the strategies developed for SC therapy. Collectively, these findings underscore that future systemic metallopharmaceutical design for SC should integrate rational ligand engineering, tumor-targeted delivery (e.g., liposomal formulations and nanocarriers), which is described in more detail below, and combination strategies addressing both cytotoxicity and redox modulation to achieve greater efficacy and reduced systemic toxicity.

To contextualize clinical translation, Table 1 compiles selected metal-based agents and regimens evaluated in skin cancer or melanoma, spanning skin-directed local approaches and systemic platinum backbones used in clinical trials. Inclusion is intended to reflect clinical evaluation rather than regulatory approval or standard-of-care status for skin cancer indications.

## 3. Innovative Approaches for Topical Therapy of Skin Cancer

Topical metallodrug delivery aims to maximize local exposure in the epidermis/upper dermis while minimizing systemic absorption. In this section, we first present how localized delivery is achieved (chemical and physical enhancers, device-assisted strategies, and PDT/photo-activation). We then summarize representative evidence and conclude with safety/manufacturing/regulatory aspects.

Topical therapies are well established in the treatment of common skin disorders, such as acne and inflammatory dermatoses, where the drug penetrates only to intradermal levels [102,137]. These examples highlight a central advantage of local drug delivery strategies: they can maximize therapeutic effects at the site of disease while limiting off-target exposure to healthy tissues. By contrast, transdermal drug delivery systems are specifically designed to enable drug molecules to permeate through the skin into systemic circulation, resulting in measurable plasma concentrations of the active substance [138].

Within oncology, topical chemotherapy represents an attractive therapeutic modality. It can be applied as a neoadjuvant intervention to reduce tumor size, thereby facilitating surgical resection and improving cosmetic outcomes by minimizing scarring [139]. Furthermore, it offers a less invasive alternative for patients in whom surgery or radiotherapy may not be feasible due to tumor location, comorbidities, or other clinical complications [80,81,92].

For metallodrugs, the success of topical administration depends not only on the choice of the active substance but also on the design of an appropriate formulation. Ideally, the drug should be able to reach the basal stratum of the epidermis, where many SCs originate, while at the same time avoiding deep penetration into the dermis. Such control over drug distribution would ensure therapeutic concentrations at the tumor site, preserve healthy tissue, and significantly reduce systemic absorption and adverse effects. To achieve this delicate balance, the use of permeation and penetration enhancers has become an important area of research. These agents facilitate the diffusion of active molecules through the epidermal layers. In topical delivery, they help distribute the drug locally within the skin, whereas in transdermal systems, they enhance systemic absorption and bioavailability [87].

In the following pages, we provide an overview of the main advances in chemical and physical permeation enhancers, drug delivery systems, and classes of multifunctional nanocarriers, all of which open new possibilities for the localized administration of metallodrugs. In parallel, the application of PDT using metallopharmaceuticals has shown promising results, reinforcing the idea that localized administration deserves renewed attention [140,141].

### 3.1. Chemical Permeation Enhancers

Chemical permeation enhancers (CPEs) are among the most widely studied strategies for overcoming the skin’s natural barrier and improving drug delivery. These molecules are typically amphiphilic and disrupt and disorganize the ordered lipid bilayers of the stratum corneum, producing nanoscale defects that facilitate drug penetration. Most studies on CPEs have investigated their incorporation into formulations, reflecting their central role in modulating cutaneous permeation [81,83,142,143,144].

For metallodrugs, the application of CPEs requires caution. CPEs may alter drug stability, bioavailability, and therapeutic efficacy. A broad variety of compounds have been investigated as CPEs, either individually or in combined formulations. These include small molecules specifically developed for this purpose, such as laurocapram (1-dodecylazacycloheptan-2-one), as well as surfactants, lipids, cyclodextrins, esters, amines, terpenes, and solvents (e.g., alcohols and sulfoxides); more recently, peptides were also considered as CPEs [81,143,145].

Despite their promise, important challenges remain. The most effective lipid-disrupting enhancers are often associated with skin irritation, which restricts their safe use in chronic applications. Furthermore, they generally exhibit limited capacity to increase the permeability of hydrophilic drugs or macromolecules, narrowing their potential clinical impact. These limitations underscore the need for ongoing research to develop safer, more versatile, and metallodrug-compatible CPEs that can be integrated into advanced topical and transdermal delivery systems.

**Figure 7:**
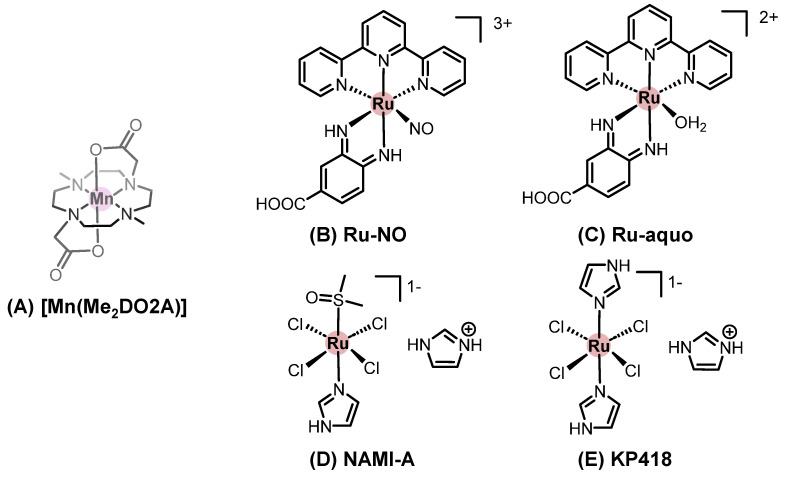
Structures of metal-based complexes evaluated in conjunction with chemical or physical permeation enhancers. (**A**) The [Mn(Me_2_DO2A)] complex, used in semisolid preparations with laureth-7 as the permeation enhancer; (**B**) Ru-NO; (**C**) Ru-aquo, used in iontophoresis protocols; (**D**) NAMI-A; (**E**) KP418, ruthenium metallodrugs evaluated in electroporation studies. Structures were drawn using ChemDraw (online version 22.2.0).

Considering metallodrugs, one example is the evaluation of different semisolid preparations on the skin penetration and permeability of [Mn(Me_2_DO2A)], where Me_2_DO2A is 4,10-dimethyl-1,4,7,10-tetraazacyclododecane-1,7-diacetate (Figure 7A). In all preparations, laureth-7 polymer (a surfactant) was used as permeation enhancer. Using human abdominal full-thickness skin samples in the Franz diffusion cell apparatus and comparing with the aqueous solution of [Mn(Me_2_DO2A)] (control group), the hydrogel allowed significant manganese permeation through the epidermis, with low manganese concentrations in both the dermis and the acceptor liquid. At the same time, the lipogel increased transdermal permeation of manganese, as evidenced by its higher concentration in the acceptor liquid. Interestingly, the emulsion gel preparation afforded the lowest manganese concentration in the skin layers (epidermis, papillary dermis, and reticular dermis) and in the acceptor liquid [57]. These results illustrate how small modifications in a formulation can impact the final application. The hydrogel preparation appeared to be useful for topical use, whereas the lipogel was a transdermal formulation and the emulgel did not release [Mn(Me_2_DO2A)] for absorption [146].

The influence of monoolein, a lipid-derived permeation enhancer, on the percutaneous penetration of cisplatin was evaluated. Using the Franz diffusion cell apparatus, aliquots of cisplatin solution (0.05%) in propylene glycol and increasing concentrations of monoolein (5 to 20%) were applied to cellulose membrane or full-thickness porcine ear skin, and the acceptor liquid samples were analyzed by high-performance liquid chromatography with diode array detection (HPLC-DAD) up to 12 h. The results indicated no effect of monoolein on the cumulative amount of cisplatin in the acceptor liquid compared to the control group (cisplatin in propylene glycol), even when the stratum corneum was previously removed. The authors concluded that the appendage pathway may be the main route to cisplatin permeation [147].

An iron(III) complex based on acrylic pressure-sensitive adhesive using modified N-[tris(hydroxymethyl)methyl]acrylamide as the ligand was developed as a transdermal patch with enhanced adhesion and cohesion properties. The permeation enhancer characteristics of this new patch material were evaluated in preclinical models for delivering clonidine (α2 adrenergic agonist), tulobuterol (β2 adrenergic agonist), ketoprofen (anti-inflammatory agent), and donepezil (acetylcholinesterase inhibitor), with promising results for both local and transdermal applications [148,149].

In parallel with CPEs, physical enhancers modulate the stratum corneum barrier to improve cutaneous deposition while constraining systemic exposure (see Section 2.1. for topical vs. transdermal definitions).

### 3.2. Physical Permeation Enhancers

Physical permeation enhancers, also called active methods, include the application of electrical stimuli (iontophoresis and electroporation), laser, or MNs to promote skin permeability. Ultrasound and radiofrequency electromagnetic waves, less commonly used in SC, were not commented in this article. All these methods have their own advantages and disadvantages, and suitability depends heavily on the specific metallodrug’s properties. Nevertheless, novel developments are projected to be more adaptable to different needs. It is worth commenting that the need for specific equipment and specialized technicians may limit the use of these procedures in outpatient and hospital settings [83,142,150].

#### 3.2.1. Iontophoresis and Electroporation

Iontophoresis involves the application of a small electrical current, usually up to 0.5 mA/cm^2^, across the skin using two electrodes to enhance the topical or transdermal delivery of the intended drug. Preferably, a charged compound is placed in the compartment containing the electrode of the same polarity, which would cause the drug to migrate in the opposite direction due to electrostatic forces [151]. Iontophoresis can also enhance the permeation of neutral species through electroosmosis induced by the electrical potential gradient [152]. The main possible drawbacks include nonspecific vasodilation, skin irritation, and burns [151,153,154].

A pilot study evaluated the use of iontophoresis and cisplatin for the treatment of 12 patients with cutaneous BCC and SCC, who were not eligible for conventional surgical therapy. Cisplatin at 1 mg/mL was incorporated in an absorbent paper patch, which was connected to the anode. No systemic cisplatin toxicity was observed for any of the patients, and minor side effects included minimal burning sensation and transient inflammatory reaction beneath the electrodes [155]. In another case report, a patient with BCC on the leg was treated with cisplatin iontophoresis [156]. The regimen consisted of four cycles of five consecutive daily applications, separated by two-week rest periods. Each treatment used 5 mL of cisplatin solution (1 mg/mL) with epinephrine hydrochloride. Post-treatment biopsy showed no evidence of BCC, confirming treatment effectiveness [156].

The use of iontophoresis was investigated to enhance the cutaneous delivery of two ruthenium(II) complexes: a nitrosyl complex [Ru(bdqi-COOH)(terpy)(NO)](PF_6_)_3_, where bdqi is 1,2 benzoquinonediimine and terpy is terpyridine, (Ru-NO), which acts as a nitric oxide (NO) donor, and its equated analogue [Ru(bdqi-COOH)(terpy)(H_2_O)](PF_6_)_2_ (Ru-aqueous) formed upon NO release (Figure 7B,C). Passive permeation of both complexes through porcine skin was limited, reflecting their high molecular weight and charge. However, iontophoresis (0.5 mA/cm^2^, 4 h) markedly improved 15-fold in the Ru-NO transport, while Ru-aqueous showed a dramatic 400-fold enhancement, with higher accumulation in the stratum corneum and viable epidermis. Notably, Ru-NO retained stability in the stratum corneum, allowing potential photo-triggered NO release after delivery, while Ru-aqueous displayed high electrotransport likely due to electroosmotic contributions. These findings highlight iontophoresis as an effective strategy to overcome skin barriers and enable localized delivery of ruthenium-based metallodrugs, with implications for treating superficial tumors and inflammatory skin conditions [157].

Following the theme of using electrical stimuli to optimize drug delivery, electroporation is another technique that relies on the use of electrical stimuli. It consists of applying short, high-voltage pulses to the skin, which creates small, transient pores in the lipid bilayers of the stratum corneum and increases drug permeability. Electroporation can be used to deliver high-molecular-weight drugs (up to 40 kDa), such as calcitonin and heparin. Still, the lack of quantitative delivery, cell death at high fields, and potential drug decomposition are the main drawbacks from this technique [75,83].

When electroporation is used to facilitate the entry of chemotherapeutic drugs (administered intravenously or intratumorally), the combined approach is known as electrochemotherapy (ECT). The ruthenium(III) metallodrugs NAMI-A and KP418 (Figure 7D,E), when delivered via electroporation, showed increased cellular accumulation and cytotoxicity against melanoma (B16-F10 cells). However, in a murine model, electrochemotherapy with KP418 did not result in any antitumor effect, in contrast to cisplatin, which presented a dose-dependent effect [158,159].

The effects of intratumoral injection of cisplatin (doses ranging from 0.25 to 2.0 mg) followed by electroporation were analyzed in four patients bearing 19 BCC, SCC, or melanoma nodules. All nodules treated with electrochemotherapy achieved a complete response after four weeks, whereas those treated only with electric pulses showed progressive disease. Controls treated with cisplatin only showed partial response or progressive disease. No systemic toxicity was observed after treatment, and cosmetic effects were minimal [129]. In another study, 10 patients with 133 melanoma nodules were enrolled in a phase II clinical trial; 82 nodules were treated with cisplatin electrochemotherapy, resulting in an objective response rate of 78%, compared to 38% with cisplatin-only treatment. The authors cited the advantages of this treatment approach as simplicity, short treatment sessions, low cisplatin doses, and minimal side effects [160].

#### 3.2.2. Laser-Assisted Drug Delivery

The goal of laser-assisted drug delivery is to compromise the skin’s barrier function by generating microscopic ablation zones using an ablative fractional CO_2_ or erbium laser [161]. Fractional beam lasers aim to damage several small areas at a specific depth within the selected target area. This approach allows for fast healing processes and a more efficient delivery of drugs. The structure of the laser beam also dictates how the light interacts with the surface irradiated according to three possible mechanisms: photothermolysis, direct ablation, or mechanical/pressure waves. The resulting increase in cutaneous porosity by this type of treatment improves the bioavailability of topically applied agents. The versatility of this strategy has gained clinical impact as a practical, highly effective, and customizable cutaneous delivery modality [162].

Two independent studies have investigated the effect of laser ablation on porcine skin to enhance cisplatin uptake [129,161,163]. In untreated skin, cisplatin largely accumulated in the superficial layers, whereas pre-treatment with an ablative fractional CO_2_ laser markedly altered drug distribution, leading to a 12-fold increase in early cisplatin penetration into deeper tissue [163]. Another study reported even greater drug accumulation, likely attributable to differences in laser parameters and cisplatin administration protocols [161]. Importantly, no systemic drug exposure was detected, highlighting the potential for localized therapy of patients with SC. Moreover, cisplatin maintained its cytotoxicity throughout treatment, as quantitative histopathologic analyses showed reduced epidermal proliferation and increased cellular apoptosis, even in healthy skin [161].

Nonetheless, the studies cited above indicate that laser-assisted drug delivery, when properly optimized, enhances the intradermal deposition of the tested agents. Collectively, these findings support laser-assisted delivery as a depth-tunable poration approach that facilitates nanocarrier traversal of the barrier, intradermal localization, and on-site payload release.

#### 3.2.3. Skin Penetration and Local Targeting

Topical metallodrug therapy hinges on two coupled processes: how carriers traverse or bypass the stratum corneum and where they are retained and release payloads within the intended cutaneous layer. Because transport across the skin barrier is largely diffusion-driven, Fick’s first law (J = −D ∂C/∂x) provides a practical framework for linking concentration gradients and lipid mobility to flux [164,165]. Three concurrent entry routes—intercellular diffusion along lipid lamellae, transcellular passage across corneocytes (uncommon for intact nanoparticles), and appendageal access via hair follicles and sweat ducts—govern depth, rate, and reservoir effects; regional follicle density (e.g., forehead > forearm) enables targeted reservoirs [166,167,168].

Carrier properties, such as hydrodynamic size, deformability, surface chemistry/ζ-potential, and partitioning into SC lipids determine whether systems remain in the SC, accumulate in follicles, or reach the viable epidermis/superficial dermis [169,170]. Stimulus-responsive designs (thermal, pH, and redox) then tune the timing and site of payload discharge; examples include thermosensitive liposomes (e.g., DPPC and 1,2-dipalmitoyl-sn-glycero-3-phosphocholine; (41–43 °C)), pH-responsive micelles (e.g., poly(2-(diisopropylamino)ethyl methacrylate), PDPA), and redox-cleavable disulfides that exploit intracellular glutathione for gold(I)/platinum(II) release [171,172,173]. Finally, patient- and site-specific factors, SC thickness, hydration, regional blood flow, and lipid composition further modulate kinetics; optical coherence tomography (OCT) can non-invasively verify in situ reach (~1–2 mm) [174,175,176].

When chemical or laser-assisted approaches cannot ensure consistent depth or dose, MNs arrays provide depth-controlled, reproducible trans-barrier access while preserving the local intent of therapy [177].

#### 3.2.4. Microneedle Technology

Microneedles are micro-sized needles arranged in arrays that penetrate the stratum corneum without causing significant pain or discomfort. MN technology has emerged as an innovative and promising approach to overcome the stratum corneum’s low permeability by creating microchannels, thereby enhancing drug permeation for SC treatment, without compromising the device’s overall safety [178,179,180]. MNs are attracting attention for their potential to improve patient compliance. Traditional methods of drug administration, such as injections or oral delivery, often face challenges such as pain, needle phobia, and variable bioavailability, and MNs overcome these limitations [181,182,183].

Several types of MNs exist, including solid, dissolving, hollow, and swelling MNs, each with unique properties and applications [184,185,186]. Solid MNs are typically made of materials like silicon or stainless steel [179,180]. Dissolving MNs are made of biodegradable polymers that dissolve after insertion into the skin, delivering the drug directly to the target site while minimizing the risk of toxicity and ensuring patient safety [187,188]. Furthermore, the use of biocompatible polymers (e.g., hyaluronic acid, poly(vinyl alcohol), and poly(lactic-co-glycolic acid)) in microneedle construction enables the loading of inorganic metal nanomaterials while maintaining favorable biocompatibility profiles, provided that polymer degradability, particle dose, and repeat-use safety are validated [94,189,190]. Hollow MNs contain a central lumen that can be used to infuse liquid drugs or vaccines [191]. Swelling (hydrogel) MNs imbibe interstitial fluid and expand after insertion, effectively enlarging transport pathways for drug penetration [94,192]. Three-dimensional (3D) printing technologies have enabled the creation of customized MN arrays [189,193], which have improved drug delivery efficiency [189,194].

One example of cisplatin-loaded MNs was the dissolvable sodium carboxymethyl-cellulose MNs prepared by molding and loaded with either cisplatin or lipid-coated cisplatin [195]. MN-mediated local delivery of lipid-coated cisplatin improved antitumor effects compared to intravenous or local subcutaneous injection of a cisplatin solution in a xenograft of head-and-neck squamous cell carcinoma (FaDu cells). Both intravenous and subcutaneous injections of cisplatin resulted in significant accumulation of cisplatin in the serum. In contrast, almost no cisplatin was observed after the MN treatment, consistent with the reduced systemic toxicity observed after MN use [195].

A second study used stereolithographic 3D printing to produce patches containing MNs, which were coated with cisplatin and a hydrophilic polymer (to increase cisplatin solubility) via inkjet printing [196]. The 3D-printed MNs showed increased skin penetration compared with those produced by the molding method. Treatment of mice bearing cutaneous SCC xenografts (A431 cell lineage) with the cisplatin-coated MNs directly on the tumor site showed modest effects (~30% reduction in tumor volume). Remarkably, skin piercing at non-tumor sites led to complete tumor regression within 5 days. The authors attributed this difference in efficacy to higher transdermal and systemic cisplatin levels when piercing occurred at non-tumor sites; in contrast, direct application to tumors may result in inefficient piercing due to heterogeneous tumor surface shapes. No platinum serum-level data were reported to confirm increased systemic exposure [196].

**Figure 8:**
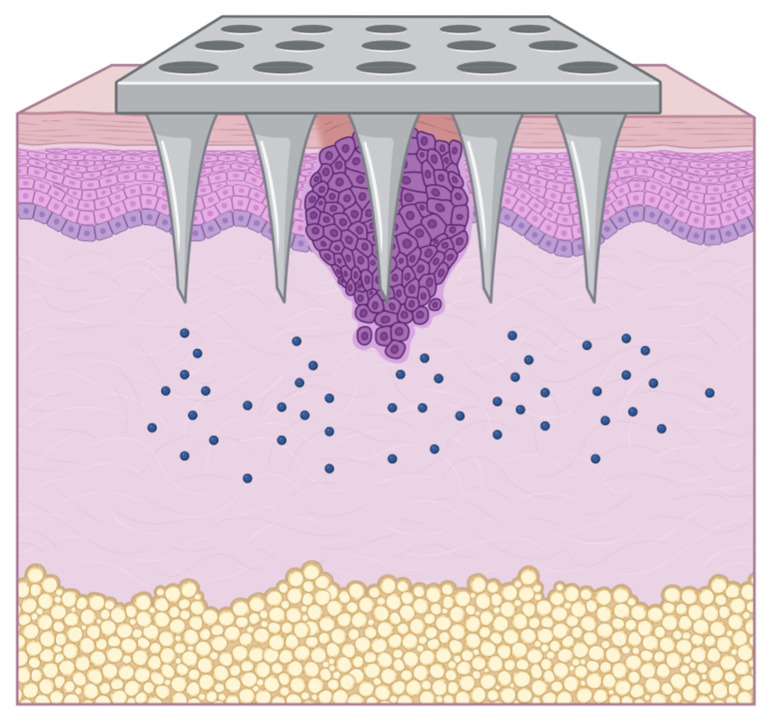
Schematic representation of metal-integrated microneedles for skin cancer therapy. Microneedles enhance transdermal delivery by passing the stratum corneum and allowing deeper penetration of therapeutic agents. Incorporation of gold nanoparticles enables photothermal activation upon laser stimulation. Zinc oxide and titanium dioxide nanoparticles contribute to antimicrobial activity and cell regeneration. Biodegradable polymeric matrices ensure controlled release and minimize systemic toxicity, improving safety and efficacy in skin cancer treatment. Created in BioRender. Van Petten, F. (2026). https://app.biorender.com/illustrations/695eb8c25fa90fdfb7aa26d0 (accessed on 10 January 2026).

The incorporation of metallic (nano)particles into MNs further enhances their therapeutic potential, offering unique functionalities such as biological activity, improved drug loading, and controlled release [181,187,193,197] (Figure 8).

Gold nanoparticles can be used to improve the stability and solubility of drugs and to enable photothermal therapy by converting light into heat, thereby enhancing drug release upon laser irradiation [194,198]. Silver nanoparticles can be applied in MNs for wound-healing applications to restore the epithelial barrier, deliver therapeutic agents, and simultaneously prevent infections, addressing two critical challenges in wound care [187]. Similarly, zinc oxide and titanium dioxide nanoparticles have been investigated for their potential to promote skin regeneration and facilitate healing [188]. All those biological applications can be leveraged for SC treatment.

The choice of metal particles and their integration method significantly influence MN properties and performance. The size, shape, and surface modification of metal particles can affect release kinetics and biocompatibility [181,183,187]. At the same time, the method of incorporating metal particles into the MN matrix, such as physical mixing or chemical bonding, also affects overall properties [182,184].

Furthermore, the stability of metal nanoparticles during fabrication and their interaction with the polymer matrix used for MNs is crucial for maintaining functional properties. The choice of polymeric materials (e.g., biocompatible hydrogels or biodegradable polymers) can significantly impact the retention and release profiles of metal particles [181,187]. Fabrication techniques such as 3D printing and micromolding enable the development of MNs with tailored architectures, thereby enabling precise control over nanoparticle distribution, orientation, and release dynamics [180,185]. Moreover, MNs equipped with sensors can provide real-time feedback on drug delivery and physiological responses, enabling personalized medicine [199,200]. This convergence of MN technology with digital health could transform how therapies are administered and monitored [186]. In addition, metal nanoparticles have been explored as imaging enhancers. For example, gold nanoparticles incorporated into MNs have shown promise as photoacoustic and computed tomography contrast agents, supporting theranostic systems capable of delivering treatment while enabling real-time tumor imaging, an avenue with potential for SC applications [185].

Given the limitations of conventional chemotherapy and radiotherapy in melanoma treatment, MNs incorporating metal-based agents have shown value for localized therapy [201]. Chen et al. (2016) developed an MN matrix composed of poly(vinyl alcohol)/polyvinylpyrrolidone embedded with lanthanum hexaboride and doxorubicin [202]. Upon near-infrared light activation, the photosensitive system produced localized hyperthermia and triggered doxorubicin release. Studies in mice showed complete eradication of superficial tumors after seven days of treatment with a polysaccharide-hyaluronic acid MN system loaded with magnetic nanoparticles (Fe_3_O_4_), dacarbazine, and indocyanine green. Hyaluronic acid enabled tumor-specific recognition, while the carbohydrate-based matrix enhanced dissolution in biological fluids and local release of both agents. The authors reported effective melanoma growth inhibition following MN-assisted phototherapy in vivo [187]. In another study targeting maxillofacial SC, a multifunctional MN system composed of iron(II) nanoparticles was engineered to release nitric oxide upon near-infrared activation. This platform induced tumor apoptosis through a NO-mediated mechanism, validated in both in vitro and in vivo models [203].

In a related approach, an MN system integrating a calcium(II)–pyrochloric acid complex for PDT was developed. Upon light activation, the system induced tumoral cell death, while calcium(II) contributed to dendritic cell maturation, potentially stimulating local antitumor immune responses [204]. A distinct example of multifunctional synergy was presented with MNs based on polyvinyl alcohol and hyaluronic acid co-loaded with calcium peroxide/copper peroxide, methyltryptophan, and disulfiram. These agents were released in response to acidic tumor microenvironments. The metal peroxides promoted ROS generation, leading to mitochondrial dysfunction and selective cytotoxicity against melanoma cells [205]. Advanced MN systems have also been explored for combining therapeutic and biosensing capabilities. These devices provide real-time feedback on drug delivery performance and treatment response [199,200,206]. This concept was expanded by combining an Au(I)–thiolate complex with a zeolitic imidazolate framework to develop a glucose-sensing biosensor incorporated into MNs. The system was proposed as a prototype for in situ monitoring of glucose metabolism in tumors, broadening its applicability to cancer diagnostics [207].

Finally, remarks, challenges, and future directions for MNs in the treatment of patients with SC should also be addressed. The development of metal particle-integrated MNs still requires refinement in key areas. Fabrication methods such as 3D printing and laser ablation must achieve uniform particle distribution and reproducible needle geometry to ensure reliable drug delivery [194]. Combining metals with biodegradable polymers could yield MNs that release drugs effectively and degrade safely, minimizing residual material. Stimulus-responsive designs triggered by temperature, pH, or other physiological cues may enable controlled, on-demand release. At the same time, integrated biosensors could enable real-time monitoring of biomarkers and site-specific drug activation, thereby improving therapeutic precision and reducing adverse effects [181]. Long-term biocompatibility and potential toxicity of metal nanoparticles are still critical concerns. The possibility of nanoparticle accumulation in tissues and its impact on healthy organ function require careful evaluation [40,181,182,183]. Furthermore, the scalability and cost effectiveness of MN fabrication need to be improved for wider clinical adoption [179,193,197].

Future research should focus on developing novel MN designs and fabrication techniques to address these challenges. The exploration of new biocompatible and biodegradable materials for MN fabrication is crucial. The development of advanced drug loading and release strategies, such as stimulus-responsive systems, can further enhance the therapeutic efficacy of metal particle-integrated MNs [181,200]. Furthermore, the integration of advanced imaging and sensing technologies into MNs can enable real-time monitoring of drug delivery and treatment response [199,200,206]. This personalized approach can improve treatment outcomes and enhance patient care. Finally, rigorous preclinical and clinical studies are essential to establish the safety and efficacy of metal particle-integrated MNs for various therapeutic applications. These studies should encompass diverse populations and consider variations in biological responses to ensure that the findings are generalizable and applicable to a broader patient demographic. Additionally, exploring regulatory pathways for the approval of metal-integrated MNs will be critical in expediting their transition from bench to bedside.

In summary, while the potential of metal particle-integrated MNs is vast, addressing the existing challenges requires a multifaceted approach that combines material innovation, smart technologies, interdisciplinary collaboration, and regulatory foresight to enhance SC care.

Beyond chemical and physical enhancers, topical drug delivery systems govern residence time, deposition depth, and photostability.

### 3.3. Drug Delivery System

Topical drug delivery systems convert a metallodrug into a local therapy by controlling cutaneous retention, depth of deposition, and photostability while limiting systemic uptake. Soft-tissue-compatible platforms, such as hydrogels/films and BNCMs, provide high water content, conformability, and chemical environments that can host metal complexes without quenching their activity. Key efficacy results for these platforms are summarized in Section 2.1; here, we focus on design principles and device–formulation choices that govern cutaneous distribution.

#### Bacterial Nanocellulose Membrane

Although the transdermal delivery of drugs can be extremely attractive, the use of BNCMs and skin patches, which release drugs onto the skin, should also be considered. Apart from poor drug utilization (only part of the applied drug dose is absorbed by the skin), the US Food & Drug Administration (FDA) and the European Medicines Agency (EMA) have encouraged the development of alternative approaches for the assessment of cutaneous bioavailability and characterization of topical formulations and their transformation into a residual film upon application to the skin [208,209]. The ease and low cost of producing skin patches are points that should also be considered.

In this context, BNC is a form of nanocellulose synthesized by certain microbial species and has garnered attention for biomedical applications. Nanocellulose itself can be categorized into several types, including bacterial nanocellulose, as well as microcrystalline, nanocrystalline, nanofibrillar, and nanowhisker forms of cellulose [210]. Among these, BNC stands out due to its purity, 3D nanofibrillar structure, and the absence of lignin, hemicellulose, and biogenic contaminants [210].

BNC is primarily produced by bacteria of the genus *Komagataeibacter*, especially *Komagataeibacter hansenii*, but other genera such as *Rhizoclonium*, *Rhizobium*, *Alcaligenes*, *Cladophora*, *Microdiction*, *Chaetomorpha*, and *Pseudomonas* have also been reported to generate cellulose under specific culture conditions [211,212,213,214]. These bacteria synthesize linear chains of glucose linked by β-1,4-glycosidic bonds, forming highly crystalline nanofibers organized into a dense 3D network. The biosynthesis process can be modulated by varying carbon and nitrogen sources in the growth medium [212,213,215].

The unique structural characteristics of BNC confer advantageous properties, including biocompatibility, biodegradability, high tensile strength (up to 2 GPa), large surface-to-volume ratio, and superior water retention capacity. These features, combined with the absence of cytotoxic components, make BNCMs an ideal scaffold for drug delivery and tissue engineering applications [216,217,218,219].

A sustained release system was developed using carboxylated bacterial cellulose (CBC), obtained by oxidation with nitrogen dioxide in a chloroform/cyclohexane mixture, as a carrier of the antitumor agent cisplatin; the results demonstrated significant potential for CBC as a drug delivery system [220]. Aquaroni et al. 2020 [221] described the antiproliferative activity of a silver(I) complex of 4-aminobenzoic acid (Ag-pABA) embedded in a BNCM. The free Ag-pABA complex showed growth inhibitory activity against human tumor cell lines, including breast cancer (MCF-7), glioblastoma (U251), multidrug-resistant ovary (NCI-ADR/RES), kidney (786-O), lung (NCI-H460), prostate (PC-3), colon (HT-29), and leukemia (K562) cells, with low cytotoxicity on non-tumor cells, such as immortalized keratinocytes (HaCaT), macrophages (J774A.1), and fibroblasts (MRC-5).

In addition, a bacterial cellulose-based adhesive membrane (BNC) carrying the silver(I)–nimesulide complex (AgNMS) was recently reported [92]. The AgNMS complex showed cytostatic effect when evaluated in vitro against tongue squamous carcinoma (SCC15), pharyngeal squamous carcinoma (FaDu), and melanoma (UACC-62) cells, with total growth inhibition (TGI) values of 67.3 µM, 107.2 µM, and 2.8 µM, respectively. For in vivo evaluation, Balb/c mice bearing verrucous carcinoma, a model analogous to human SCC, were topically treated with AgNMS incorporated into a BNC-based adhesive dressing. The treatment resulted in up to 100% tumor size reduction along with no signs of local or systemic toxicity. Notably, the BNC membrane provided physicochemical stabilization of the AgNMS complex, including enhanced resistance to photodegradation, making it a promising platform for topical therapeutic applications. Moreover, in vitro evaluation demonstrated that the transdermal device maintained AgNMS release for up to 216 h without interruption [92].

Nanocrystalline cellulose nanoparticles (CNC NPs) have also been studied for drug delivery. Imlimthan (2021) showed that treatment with lutetium-labeled CNCs loaded with vemurafenib ([177Lu]Lu-CNC-V NPs) doubled the survival time in an in vivo metastatic melanoma mouse model compared to the control group [222].

Although bacterial nanocellulose has been extensively studied as a therapeutic carrier [92,190,216,221,223,224,225,226,227], its application as a support for the localized release of metal complexes in tumor treatment remains relatively unexplored. This highlights the potential of BNC-based membranes as a strategic platform for localized therapies in SC, particularly when combined with cytotoxic or photoreactive metal complexes. Future research should focus on integrating BNC systems with permeation enhancers or physical delivery techniques to optimize cutaneous bioavailability and enhance therapeutic precision.

### 3.4. Characterization, Quality Control, and Stability of Nanocarriers

#### 3.4.1. Rationale and Current Challenges

Metallopharmaceuticals, including gold, platinum, and ruthenium complexes, have shown remarkable anticancer potential through redox modulation, DNA binding, and controlled generation of ROS [103,227]. These properties make them particularly attractive against SCs, including SCC and melanoma. However, despite promising in vitro and preclinical results, their clinical translation remains limited due to physicochemical and biological barriers [228,229,230].

A central challenge is the intrinsic instability of metal complexes in physiological environments [231]. Rapid hydrolysis, ligand exchange, and redox reactions can lead to premature deactivation or transformation of the active species [86,232]. Interactions with sulfur-containing biomolecules, such as glutathione and metallothioneins, and competition with endogenous metal ions further disrupt the integrity of the complex [230,233]. These reactions occur not only during systemic circulation but also within the skin microenvironment, where the acidic stratum corneum contrasts with the neutral viable epidermis and dermis, creating pH gradients that accelerate degradation [7,178,234].

In topical applications, additional limitations arise. Poor aqueous solubility hinders drug dispersion in hydrophilic carriers, and aggregation of hydrophobic complexes can reduce permeation and bioavailability while increasing local irritation and systemic toxicity [178,230]. Conventional formulations such as creams or gels often fail to maintain chemical integrity and therapeutic activity during penetration and skin residence. Therefore, multifunctional nanocarriers, which are considered strategic advanced delivery systems capable of stabilizing metallodrugs, are essential to unlock their full therapeutic potential [229,235].

#### 3.4.2. Classes of Multifunctional Nanocarriers

**Figure 9:**
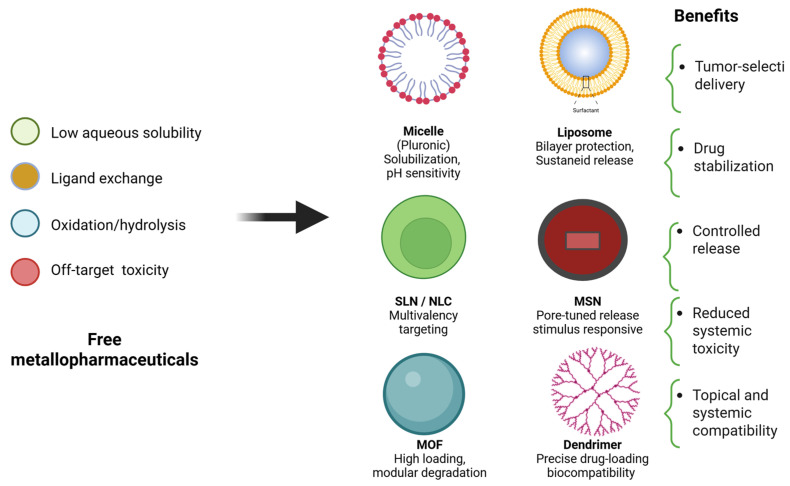
Nanocarrier strategies to stabilize and deliver metallopharmaceuticals for skin cancer. The schematic illustrates key challenges of free metallopharmaceuticals, including low water solubility, ligand exchange, chemical degradation (oxidation/hydrolysis), and off-target toxicity (left panel), and highlights nanocarrier platforms (center panel) designed to overcome these issues through protective encapsulation and controlled delivery. These systems comprise Pluronic^®^-based micelles (pH-sensitive delivery, drug stabilization, controlled release, reduced systemic toxicity, and compatibility with topical or systemic administration), liposomes (bilayer protection with sustained release), solid lipid nanoparticles/nanostructured lipid carriers [SLNs/NLCs] (multivalent targeting and enhanced stability), mesoporous silica nanoparticles [MSNs] (pore-tuned and stimulus-responsive release), metal–organic frameworks [MOFs] (high loading capacity and modular degradation), and dendrimers (precise drug loading with high biocompatibility). These multifunctional platforms confer key therapeutic advantages (right panel), such as tumor-selective delivery, improved pharmacokinetics, increased chemical stability, and reduced local/systemic toxicity. Image created in BioRender. Van Petten, F. (2026). https://app.biorender.com/illustrations/695f1613f9a4ae6cd56b1e48 (accessed on 10 January 2026).

Multifunctional nanocarriers can stabilize coordination bonds against premature hydrolysis and ligand exchange, improving aqueous solubility, reducing aggregation, and enabling controlled release in targeted tissues. By providing protective microenvironments, nanocarriers can preserve drug integrity from manufacturing through administration and improve pharmacokinetic profiles and therapeutic selectivity [201,229,230]. These systems can be broadly categorized into polymeric micelles, lipid-based carriers (such as liposomes and nanostructured lipid carriers), inorganic matrices (such mesoporous silica nanoparticles and metal-organic frameworks), and dendrimers, offering advantages in drug loading capacity, stability enhancement, release kinetics, and biological interactions. The most widely studied strategies and their therapeutic implications in SC are illustrated in Figure 9, which contrast the instability and off-target toxicity of free metallodrugs with the protective encapsulation and controlled delivery achieved by platforms.

#### 3.4.3. Polymeric Micelles

Polymeric micelles form hydrophobic cores capable of solubilizing poorly water-soluble metal complexes, thereby preventing their aggregation and premature degradation. Pluronic^®^ triblock copolymers (poly(ethylene glycol)-block-poly(propylene glycol)-block-poly(ethylene glycol), PEG-PPG-PEG) self-assemble above their critical micelle concentration (CMC) and/or critical micellization temperature (CMT), generating a PPG hydrophobic core and a PEG hydrophilic corona [236]. This strategy was efficient to preserve the stability and bioactivity of ruthenium(III) complexes with formulas [Ru(dtc)_3_], α-[Ru_2_(dtc)_5_]Cl, and β-[Ru_2_(dtc)_5_]Cl, where dtc signifies the dithiocarbamate ligands *N,N*-dimethyl dithiocarbamate, pyrrolidyl dithiocarbamate, sarcosyl-alkyl-ester dithiocarbamate, and carbazolyl dithiocarbamate under physiological conditions in tumor cell models [237]. Moreover, a copper(II) complex [CuCl_2_(4′-(4′-meta-methoxy-phenyl)-2,2′: 6′,2″-terpyridine)] maintained high stability over 96 h in simulated biological media when encapsulated, in contrast to the rapid precipitation of the free form consistently [238]. Overall, Pluronic^®^ F127 micelles increase solubilization and stabilization of metal complexes [238].

Carbohydrate-functionalized Pluronic^®^ F127 micelles were engineered to enhance cancer-selective drug delivery. Ruthenium(II) and copper(II) dithiocarbamate complexes of formulas β-[Ru_2_(Pipedtc)_5_]Cl and [Cu(ProOMedtc)_2_], where Pipedtc is piperidine dithiocarbamate and ProOMedtc is *L*-proline methyl ester dithiocarbamate, were encapsulated within the hydrophobic micellar core and evaluated in vitro against aggressive human cancer cell lines. The formulations were thoroughly characterized by dynamic light scattering (DLS) and transmission electron microscopy (TEM), while mechanistic studies using confocal laser scanning microscopy and xCELLigence confirmed enhanced cellular uptake and potent cytotoxicity [239]. Collectively, these results highlight carbohydrate-functionalized Pluronic^®^ micelles as a promising nanoplatform for the selective delivery of metallodrugs in cancer therapy.

#### 3.4.4. Lipid-Based Systems

Liposomes are the most established and clinically advanced nanocarriers for metallodrugs [236]. Structurally composed of one or more concentric phospholipid bilayers surrounding an aqueous core, they create spatially distinct hydrophilic and lipophilic domains. This architectural versatility allows for the encapsulation of a wide range of metal complexes with different physicochemical properties: hydrophilic compounds can be entrapped in the aqueous lumen, amphiphilic species may partition at the bilayer interface, and lipophilic metal complexes can be incorporated into the hydrophobic membrane core. Such compartmentalization not only improves biocompatibility, membrane fusion, and cellular uptake but also protects labile complexes from hydrolysis, redox reactions, and destabilizing ligand exchange with biological nucleophiles [236,240].

Experimental evidence highlights these advantages. Amphiphilic ruthenium(II) complexes were successfully encapsulated into 1-palmitoyl-2-oleoyl-sn-glycero-3-phosphocholine (POPC) liposomes, demonstrating long-term colloidal stability exceeding 30 days and enhanced antiproliferative activity compared to the free drug [240]. Beyond liposomes, other lipid-based platforms such as solid lipid nanoparticles (SLNs) and nanostructured lipid carriers (NLCs) have emerged as second-generation systems with superior physicochemical robustness. Their solid or semi-solid lipid matrices keep metallodrugs dispersed, reduce premature leakage during storage, and enable controlled release at tumor sites [241]. Improved cutaneous delivery has been demonstrated by enhanced stratum corneum penetration and follicular targeting with lipidic nanocarriers [242,243,244] and by greater topical efficacy in skin-cancer models using nanoformulations [242]. Together, these data support lipid-based nanocarriers as versatile, clinically relevant platforms for stabilizing and delivering metal complexes in cutaneous oncology.

**Figure 10:**
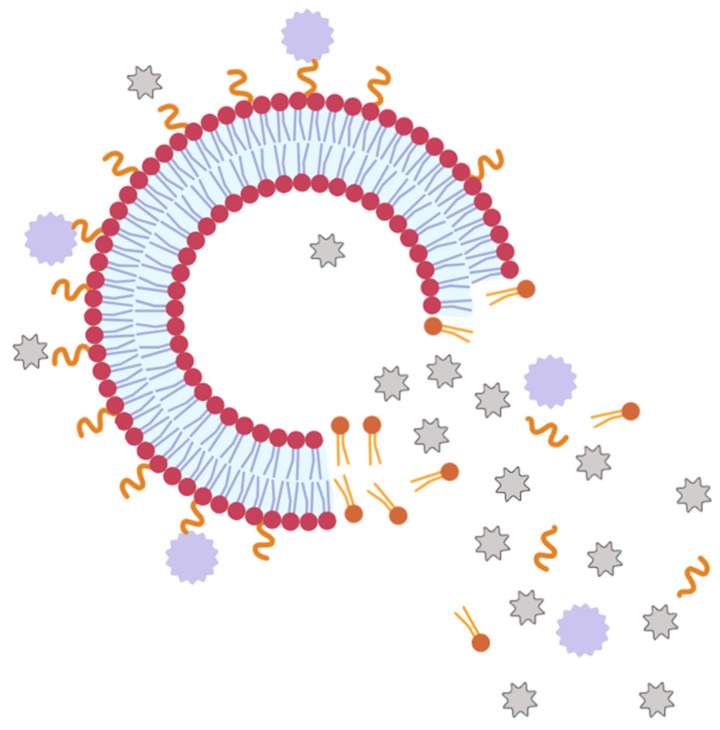
Schematic representation of liposomes as nanocarriers for metallopharmaceuticals. Depending on their chemical nature, metal complexes can be incorporated into the aqueous core, embedded within the lipid bilayer, or associated at the membrane interface. This versatility enables improved stability, controlled release, and enhanced bioavailability for skin cancer therapy. Created in BioRender. Van Petten, F. (2026). https://app.biorender.com/illustrations/69655dd2516fd5738cc32a81 (accessed on 10 January 2026).

A schematic representation of liposomal architecture, illustrating the possible localization of metallopharmaceuticals within the aqueous core, lipid bilayer, or membrane interface, is presented in Figure 10. This figure emphasizes how liposomal structural versatility underpins their ability to improve stability, regulate release, and increase bioavailability of metal-based drugs in SC.

#### 3.4.5. Dendrimers and Nanogels

Dendrimers are highly branched macromolecules with precise architecture that allow for exceptional control over drug loading, surface functionality, and release kinetics. Poly(amidoamine) (PAMAM) dendrimers provide multiple coordination sites for metal complexes, steric protection against destabilizing biomolecules, and the potential for theragnostic integration via co-loading of imaging agents. Recent reviews emphasize how PAMAM dendrimers can be engineered to enhance the stability of metallodrugs, improve targeted tumor delivery, and integrate diagnostic capabilities for real-time monitoring of therapeutic response [245,246]. Together, these reviews reinforce the translational value of PAMAM dendrimers as multifunctional carriers for both diagnostic and therapeutic applications [247].

Beyond conceptual advances, preclinical studies have shown that dendrimers can substantially improve the therapeutic profile of platinum complexes. For instance, PAMAM dendrimer conjugates of platinum(IV) demonstrated five-fold higher tumor accumulation in murine xenograft models compared to the free drug while reducing nephrotoxicity by over 60% [247,248]. Such results underscore the translational potential of dendrimers as multifunctional nanocarriers that simultaneously stabilize labile metal complexes, enhance tumor selectivity, and mitigate systemic toxicity. Complementary engineering strategies to optimize PAMAM dendrimers, including reducing systemic toxicity, enhancing stimulus-responsive release, and improving tumor targeting, are also described in the reviews [245,248].

### 3.5. Stability of Metallopharmaceuticals in Transdermal Systems

The principal challenge in transdermal metallodrug delivery is preserving chemical integrity during in-skin residence and barrier transit. Metal complexes are particularly susceptible to hydrolysis and ligand exchange with endogenous thiols (e.g., glutathione, cysteine, and metallothioneins), which can prematurely inactivate the drug [249,250]. In contrast, ruthenium(III) complexes resist direct hydrolysis but undergo bioreduction to ruthenium(II) in the presence of cellular reductants, modulating bioactivation in viable tissues [251]. The skin tumor microenvironment with an acidic stratum corneum overlying near-neutral viable layers, local oxidative stress and enzymes, UV-induced ROS, and a mildly acidic extracellular pH further stresses stability [252,253].

Several formulation strategies counter these liabilities: polymeric micelles maintain solubility and limit precipitation [236], while liposomes (phosphatidylcholine/cholesterol) can shield coordination bonds [254]; nanostructured lipid carriers reduce molecular mobility [241]; and dendrimers add steric shielding while enabling controlled release [255]. Biopolymer matrices such BNCMs have also shown utility, providing strong bioadhesion and sustained, localized release (~72–96 h) for labile metal systems [220,221].

In practice, clinical performance depends on achieving a delicate balance between protection and release. The system must provide sufficient encapsulation to prevent premature degradation during transit through the stratum corneum yet still permit efficient payload discharge in viable tissue. This balance is often tuned by lipid composition, stabilizing excipients, and, when appropriate, stimulus-responsive triggers [166,167], including light-activated schemes used in PDT [256,257].

### 3.6. Photodynamic Therapy

Photodynamic therapy has emerged as a minimally invasive therapeutic strategy alternative to surgery, particularly for superficial lesions and for patients unfit for surgical procedures [258,259,260].

The mechanism of action of PDT involves the activation of a photosensitizer (PS) upon light exposure. Once activated, the PS generates reactive oxygen species (ROS) via two main pathways: type I reactions (electron transfer, leading to free radicals) or type II reactions (energy transfer to oxygen, producing singlet oxygen); these species are cytotoxic and induce apoptosis, necrosis, or autophagy. PDT may also elicit antitumor immune responses, enhancing therapeutic outcomes. Due to the distinct pharmacokinetics of PS molecules in tumor versus normal cells, PDT offers selectivity, as tumor cells tend to retain higher concentrations of the PS [258,261,262].

Regarding the development of PS, molecules are broadly classified into three generations: first-generation porphyrin derivatives, second-generation porphyrin precursors, and third-generation chlorins and bacteriochlorins, which were developed to improve tissue selectivity and excitation wavelength for deeper tumors [256,263]. PS molecules have varying potential for producing ROS through type I or II reactions. They can be administered through different methods and absorb various wavelengths, making them suitable for targeting diverse tissue, tumor cell characteristics, and volumes. Depending on the specific approach, these molecules may be administered either topically or intravenously [264].

**Figure 11:**
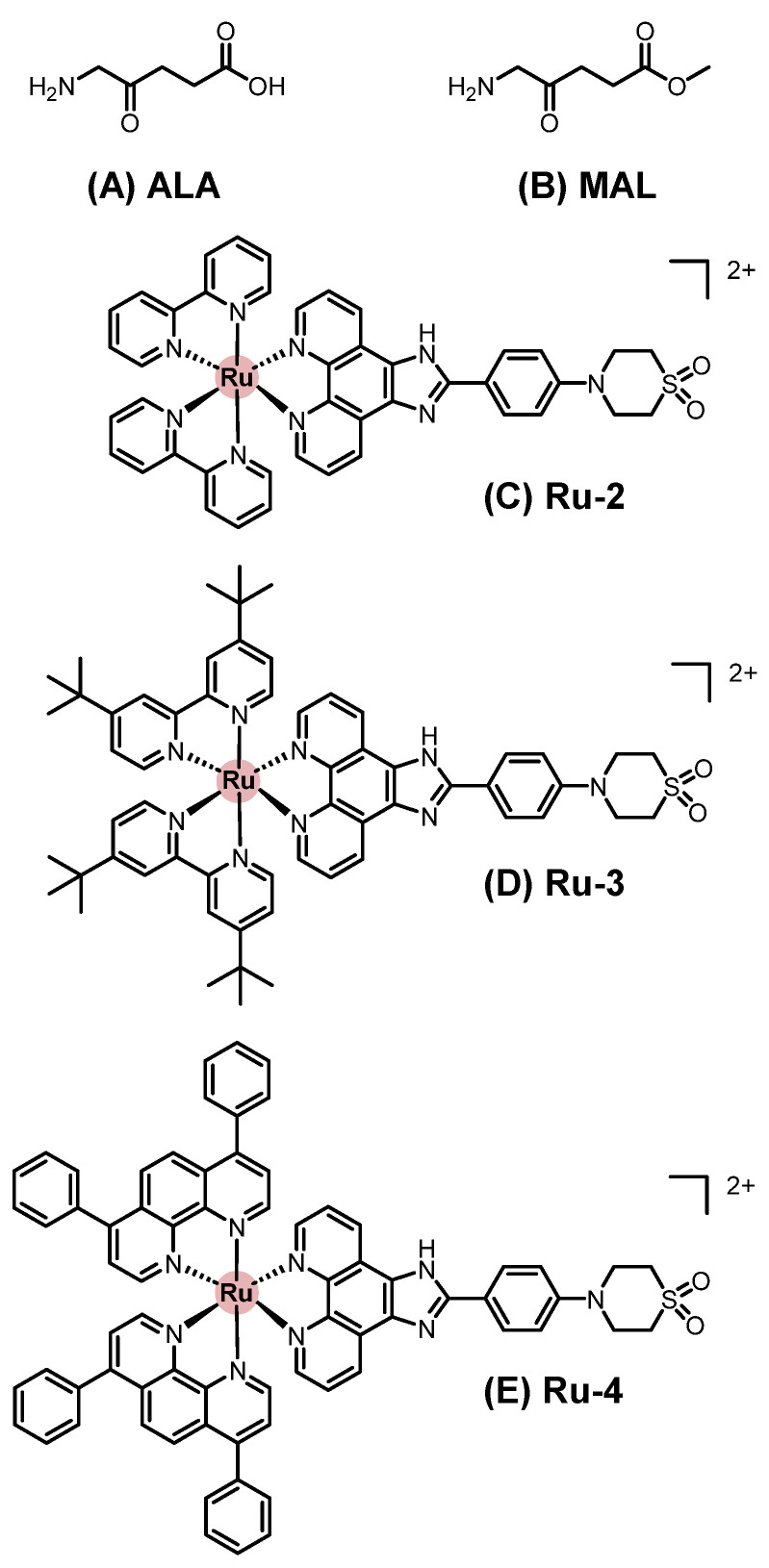
Structural representation of compounds used in PDT. (**A**) Aminolevulinic acid (ALA), (**B**) methyl aminolevulinate (MAL), and (**C**–**E**) ruthenium(II) complexes (Ru-2, Ru-3, and Ru-4) have been explored for light-activated antitumor therapies.

Topical PDT is based on the delivery of aminolevulinic acid (ALA) (Figure 11A) or methyl aminolevulinate (MAL) (Figure 11B) through a transdermal cream formulation [188,265]. The systemic administration is performed using the intravenous injection of porphyrin or chlorin compounds [266]. The possibility of using topical PDT based on ALA, a pro-drug to induce the production of an endogenous PS, increased the PDT application, mainly due to the lack of systemic photosensitivity.

PDT was approved for the treatment of several cutaneous disorders, such as psoriasis, acne, and warts [258]. Topical PDT has also been used in antimicrobial and cosmetic therapies. The cosmetic application is performed using ALA-PDT but with lower ALA concentration, lower pre-light time, and lower irradiance and fluence. This application aims to induce a superficial cell death and take advantage of the improved cosmetic response of the photodynamic action. The induced production of cytokines and growth factors are described as the main effects of dermal remodeling and PDT rejuvenation [267].

PDT has also been used with antimicrobial purposes. The antimicrobial PDT is based on the same mechanism of action but with the microorganisms as the target cells. In this case, the PS, mostly methylene blue and curcumin, is topically applied to the infected skin lesion, and after an incubation time, the lesion is illuminated. The ROS induce damage mainly to the biomolecules present at the cell wall and membrane, resulting in pathogen inactivation. The main applications of antimicrobial PDT for dermatology are for the treatment of onychomycosis [268,269], infected wounds, and acne [270], but other uses of PDT for skin infections have also been reported, such as for sporotrichosis [271] and pythiosis [272,273]. With the ever-growing search for more efficient PSs, in the early 2020s, the exploration of metallopharmaceuticals has become prominent, particularly those involving ruthenium and platinum complexes, aimed at combining cytotoxic and photodynamic effects.

In oncology, PDT has been indicated particularly for actinic keratoses (AKs), seen as cutaneous premalignant lesions, cutaneous lymphoma [274], and NMSC [275], which is the most common type of cancer worldwide. Application protocols vary in types, concentrations, and routes of administration of PSs, as well as the light sources used, leading to different levels of efficacy.

Topical PDT is limited in treating superficial lesions, such as AK, and NMSC. According to the American Academy of Dermatology and the European Dermatology Guidelines, topical PDT with one or two sessions, usually using ALA or MAL, has been indicated for both conditions. These guidelines reported different protocols that have been established for topical PDT, with incubation times ranging from 1 to 5 h and irradiation with 630 nm ranging from 37 to 75 J/cm^2^ [276,277]. Superficial BCC is the main indication for ALA-PDT, which is of special interest in cases where the tumor site is on the face or there are several lesions, where surgical resection may highly compromise the aesthetics and functionality of tumor carriers. Eyelids, noses, and ears are facial areas where PDT, topical or systemically applied, does not compromise function after tissue healing [278].

A multicenter study was conducted in Brazil, and professionals from 72 centers were trained to administer PDT for patients with BCC. The protocol adopted was a 3-h incubation with 20% MAL cream, followed by irradiation at 630 nm with a fluence of 150 J/cm^2^, in two sessions with a 1-week interval. The complete response rate for superficial BCC was around 90% and, for nodular lesions, 60–70% [261,279]. However, topical treatment has been recommended to treat BCC lesions up to 2 mm thick due to the limited penetration of the cream throughout the lesion [280].

In Brazil, PDT has been widely adopted, with innovations in the fluorescence-guided application procedure, enabling real-time feedback on treatment evolution to be obtained [280]. This has enabled relatively high success rates and led to its approval by public health authorities [262]. Given its increasing relevance, further improvements are being pursued to reduce incubation time and enhance tissue penetration, aiming to increase the extent of necrosis and reduce recurrence. To achieve this, the development of soluble MNs, metabolic stimulation strategies, and advanced light engineering systems are underway. Clinical projections expect success rates above 97% with substantial long-term recurrence reduction [281]. These recent advances in PDT delivery and optimization highlight the growing relevance of integrating metal-based agents and chemical permeation strategies to improve localized therapeutic outcomes, particularly in SCC.

Thicker BCC and SCC tumors may be indicated for PDT, but only when using systemic PS. The main disadvantage of the systemic PDT is the prolonged skin and eye photosensitivity, which lasts 4–6 weeks with porphyrin and 1 week with chlorin. When compared with surgical resection, PDT showed improved cosmetic response, and with a one-session protocol, a similar complete response was seen for NMSC [280,281]. Another relevant advantage of PDT is the lower risk of contamination and further infection at the treated site. For the treatment of more massive tumors that require deeper penetration, porphyrin derivatives, such as Photofrin^®^, and chlorins can be used and administered intravenously. These options provide greater penetration depth and enhanced effectiveness in advanced tumors [280,282]. In cases of SCC in the head and neck, complete response has been observed in 60% to 80% of cases. In these cases, the standard treatment surgery, can often be unfeasible for the patient, especially in areas of the face, due to aesthetic and functional damage [257,283].

Metallopharmaceuticals potentially enhance PDT efficacy. In this area, metallodrugs have been developed and improved to allow other treatments such as immunotherapy, chemotherapy, or radiotherapy to be applied together with PDT due to their photochemical and photophysical properties, thus helping achieve greater treatment efficacy in the clearance of tumors [267].

Ruthenium-based compounds have high photostability and strong light absorption within the therapeutic window and may act simultaneously as PSs and as direct sources of cytotoxicity. They selectively accumulate in tumor tissues generate ROS when irradiated [284,285].

In a recent study, three ruthenium(II) complexes, Ru-2, Ru-3, and Ru-4, were synthesized [286]. The representative chemical structures of the ruthenium(II) complexes are shown in Figure 11C–E, with the composition [Ru(L)_2_(dotmp-pip)_2_], where L = bipy, 4,7-diphenyl-1,10-phenanthroline (dipphen), or 4,4′-di-tert-butyl-2,2′-bipyridine (tbubpy), and dotmp-pip is 2-(4-(1,1ioxidothiomorpholino)phenyl)imidazo [4,5f][1,11]-phenanthroline. Ru-3 was encapsulated in liposomes and formulated into a cream for the treatment of SC as a transdermal drug delivery system (LipoRu). Accelerated diffusion capacity and a remarkable antitumor effect with low toxicity were observed when LipoRu cream was topically administered to A375 tumor-bearing mice (A375) via a PDT approach. Although the study was performed in a melanoma model, the formulation strategy involving ruthenium(II) complexes and liposomal carriers represents a promising topical delivery approach that could be translated to other skin malignancies [284,285,286].

Platinum-based agents have been investigated for their potential to cause DNA damage concomitant with photodynamic action, and some of them release cytotoxic platinum species upon light irradiation, thereby increasing localized tumor damage [285,287]. In addition, metallodrugs can be used as markers for tumor tracking via imaging techniques such as single-photon emission computed tomography, positron emission tomography, and magnetic resonance imaging [247]. These findings highlight the potential of metallopharmaceuticals when incorporated into delivery platforms, reinforcing the need for preclinical studies specifically targeting BCC and SCC models [247,287].

Other examples, such as the iridium-based complex containing the (4,15-bis [4-(N,N-diphenylamino)phenyl] [1,2,5]thiadiazolo-[3,4-i]dipyrido[a,c]phenazine) ligand and titanium oxide nanoparticles, have been investigated to provide strong absorption in the near-infrared wavelengths, thereby increasing light penetration in tissues. They result in efficient ROS generation, representing a promising strategy for improving PDT outcomes in more complex lesions [199,288]. Gold and silver nanoparticles have been employed as PS agents. Their metallic nature also enables localized surface plasmon resonance effects, which amplify the photodynamic response, as well as other optical effects that may contribute to treatment monitoring, such as luminescence generation [289,290].

**Figure 12:**
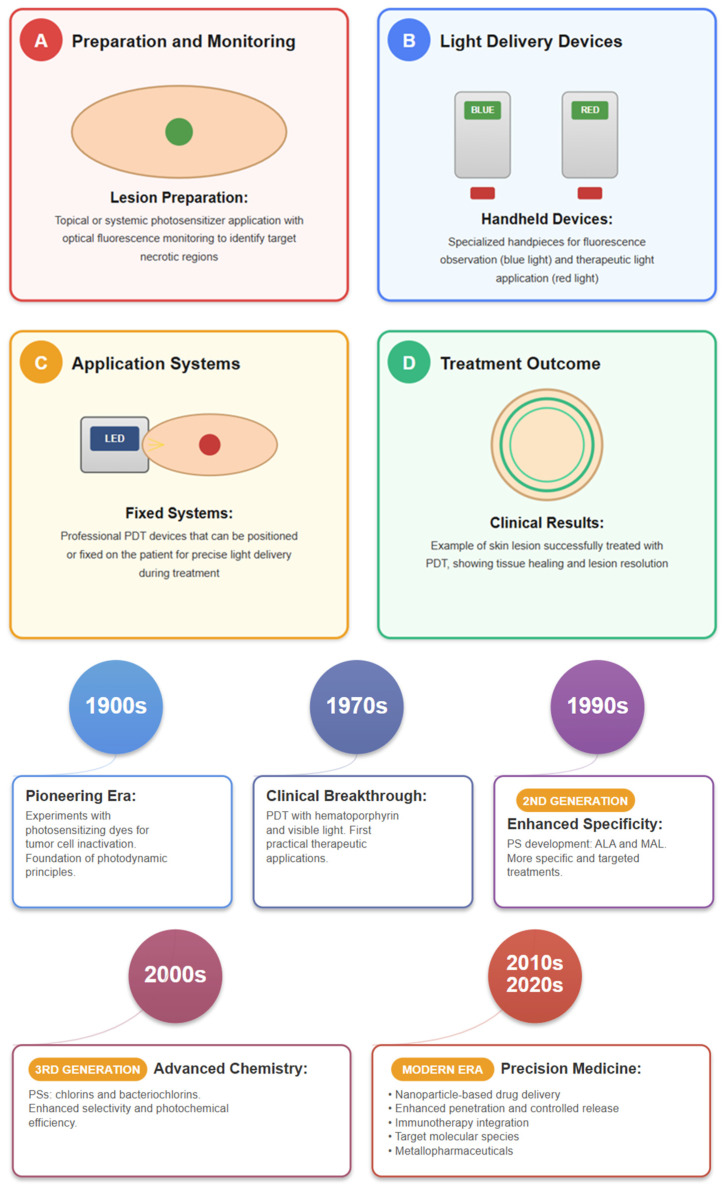
Photodynamic therapy from foundational experiments to advanced clinical applications and treatment workflows. The timeline illustrates key milestones: pioneering studies using photosensitizing dyes for tumor cell inactivation in the early 1900s; clinical breakthrough in the 1970s with hematoporphyrin and visible light; development of second-generation photosensitizers such as aminolevulinic acid (ALA) and methyl aminolevulinate (MAL) in the 1990s, offering enhanced specificity; third-generation chlorins and bacteriochlorins in the 2000s with improved selectivity and photochemical efficiency; and, from the 2010s onward, the integration of precision medicine strategies, including the use of metallopharmaceuticals. On the right, the PDT clinical procedure is schematically represented: (**A**) lesion preparation via topical or systemic photosensitizer application and fluorescence monitoring for target tissue localization; (**B**) handheld light delivery devices for diagnostic observation (blue light) and therapeutic irradiation (red light); (**C**) fixed application systems enabling accurate and reproducible light exposure; and (**D**) typical treatment outcome demonstrating lesion resolution and tissue healing. AI-generated illustration using Claude; edited and finalized by the authors.

**Figure 13:**
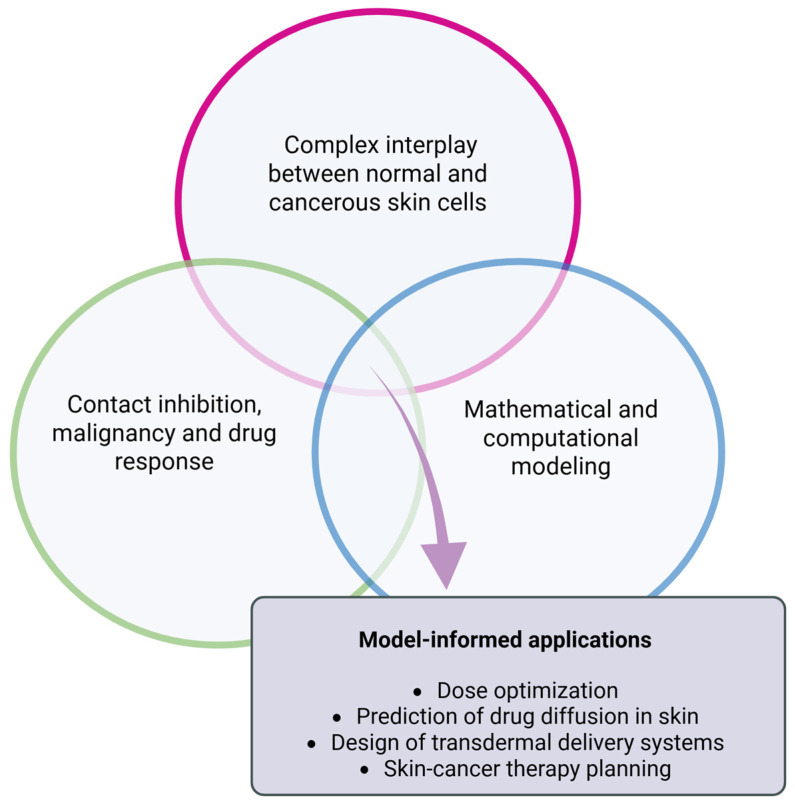
Integration of possible key factors in modeling skin cancer therapies. Each circle represents a major component: the complex interplay between normal and malignant skin cells, contact inhibition, and mathematical and computational approaches. The intersection of all three highlights potential outcomes of these interactions, including refinement of drug dosing, predictions of drug diffusion, and the design of transdermal therapies in skin cancer. Created in BioRender. Van Petten, F. (2026). https://app.biorender.com/illustrations/69655dc20c029b1a6ac86050 (accessed on 10 January 2026).

A timeline of advancements in PDT, emphasizing its evolution from initial exploratory studies in the early 1900s to contemporary technological innovations, is presented in Figure 12 [266]. Notable milestones include the introduction of hematoporphyrin-based therapy in the 1970s, which served as a precursor to the subsequent approval of second- and third-generation PSs [263,276]. The timeline also delineates significant progress in nanoparticle-based drug delivery systems, which have enhanced the precision of therapeutic interventions [260]. In recent years, PDT has undergone further evolution through its integration with immunotherapy and the development of metallopharmaceuticals [284,291], leading to improved treatment efficacy and the expansion of its clinical applications. In addition to highlighting the chronological development, Figure 13 also illustrates the components of the clinical procedure involved in PDT.

### 3.7. Safety, Manufacturing Quality, and Regulatory Considerations

For topical metallodrug patches and BNC-based devices, clinical translation hinges on three pillars: (i) dermal safety, using standardized protocols for irritation, sensitization, repeated-dose effects, and phototoxicity when photoactive centers are present (e.g., OECD TG 404 and OECD TG 429; ISO 10993-10 and ISO 10993-23; OECD TG 432; OECD, 2015; OECD, 2010; ISO, 2010; ISO, 2021; OECD, 2019); (ii) local deposition and persistence, verified by histopathology and elemental mapping (ICP-MS/LA-ICP-MS) to exclude chronic inflammation or granuloma from residual metal [292,293]; and (iii) manufacturing quality, including content uniformity and dose per area, adhesive performance–peel, extractables–leachables, endotoxin—bioburden control, residual solvents, and stability (chemical/photo) under ICH/ISO-aligned conditions (USP, 2019; FDA, 2019; ISO, 2020; ISO, 2018; ICH, 2003; ICH, 1996) [294,295]. When systemic exposure is intended to be negligible, exploratory toxicokinetics (TK) should confirm only trace plasma levels and absence of organ accumulation; for triggered systems (e.g., mild-hyperthermia liposomes), stimulus safety and release robustness must also be demonstrated under conditions of use. Together, these requirements define the constraints used in Section 5 for model-based dose and device design [294].

## 4. Mathematical and Computational Approaches for Skin Penetration

The mammalian stratum corneum performs many functions essential to the skin’s protective role [296]. These functions serve as a key barrier to achieving effective transdermal drug delivery [297,298]. Overcoming this natural barrier and maintaining the drug at the targeted skin layer pose significant technical challenges in this field [298]. To facilitate skin penetration, certain physical factors must be present, regardless of the metallic properties of the pharmaceutical agent. This indicates that computational and mathematical modeling can be applied broadly in this context.

Although the processes governing the skin penetration of chemicals and nanoparticles may differ [298], the physical size of the delivered agent is always significant. In terms of depth, the stratum corneum provides a highly effective barrier to penetration at a thickness of only 15–20 µm (for comparison, a red blood cell has a typical diameter of ∼10 µm) [299]. Given this thickness, one of the first significant contributions of mathematical modeling in this field was the establishment of a threshold molecular weight (Da) as a typical molecular weight for transdermal agents. Below this molecular weight, skin penetration is considered feasible.

This determination was historically made by analyzing a simple ordinary differential equation model, under the assumption that the cumulative mass of a diffusant passing through the membrane per unit area reaches a steady state, represented by a straight line [300].

Due to the skin structure, it is critical to note that there are three pathways for a penetrant to reach the living tissue: through eccrine sweat ducts, through hair follicles with attached sebaceous glands, or through the continuous stratum corneum between these appendages [301]. The route of the appendages is generally not significant for drug delivery, as their surface area accounts for only 0.1% of the total area available for transport [299,301]. Therefore, the primary pathways for drug penetration are the intercellular and transcellular routes within the continuous stratum corneum.

In practice, the distinction between intercellular and transcellular routes is not straightforward. Still, they have historically been described using the “brick and mortar” heterogeneous structural model proposed by Michaels et al. in 1975 [302]. In their paper, the authors provide mathematical treatment of skin permeation measurements to analyze their experimental results. This progress continued in the same decade, with both theoretical [303] and in vivo experimental descriptions [303] suggesting that the intercellular route was important. Mathematically, this came to light using Fick’s diffusion law, with slow interfacial transfer kinetics across the different layers of the stratum corneum. Using the overlapping brick model, the effective diffusion path length can reach 300–350 µm, which is significantly greater than the 20 µm stratum corneum thickness [303].

Many other quantitative and mathematical approaches have been applied to transdermal drug delivery. These can be essentially categorized into two main groups: (1) empirical models, which are strictly based on experimental data but not on physical principles (e.g., machine learning models or purely statistical models such as multiple linear regression), and (2) mechanistic models [304], which, in turn, can be classified according to the spatial scale and physical structure involved in their description. The modeling of drug permeation by Fickian diffusion is one of the most prominent mathematical descriptions underlying these models.

Alternative modeling approaches include the use of compartments representing different sites along the skin depth to which the chemo agent can diffuse, from its historical origins to classic compartmental analyses, and other refined approximations of diffusion models in skin drug transport [305]. Other mathematical contributions to SC therapies include modeling MN-mediated transdermal delivery [306] and optimizing dosimetry in photomedicine [307], where an integrated mathematical oncology approach may prove to be a promising tool in the fight against cancer.

On the basic research side, it is worth noting that one of the most basic uses of mathematics in drug discovery is the direct calculation of the half-maximal inhibitory concentration (IC_50_), which is a key metric for the potency of anticancer agents, including metallopharmaceuticals for SC [87]. Although it may sound too simple, the proper determination of IC50 values and their uncertainties requires caution in model fitting. For that, this need still demands the development of state-of-the-art statistical methodologies and mathematical tools such as those coded in the valuable (version 2.0.5; https://cran.r-project.org/package=drda, accessed on 10 December 2026) [308].

Moreover, mathematical models serve practical purposes and provide valuable theoretical insights that can be tested in real-world settings. Sometimes, these models are inspired by experimental data, even if they are not directly based on them. For example, a study employing ordinary differential equations supported the hypothesis that fibroblasts contribute to melanoma growth and drug resistance [309]. Notably, the recent literature demonstrates that cancer-associated fibroblasts contribute to resistance to anticancer therapies [310,311,312]. This example highlights the importance of applied mathematics in illuminating and raising thought-provoking questions for future research on SC.

The complex interplay between normal and malignant cells is increasingly being explored through mathematical and computational approaches. Most mathematical models of tumor growth often neglect the explicit role of surrounding healthy tissues, yet these interactions are central to understanding both tumor progression and therapeutic response. The concept of contact inhibition, first described as a hallmark of normal cell regulation whose loss was linked to malignant transformation [313], laid the foundation for later experimental and theoretical studies. Building on this, more recent work has shown that contact inhibition is a key mechanism that orchestrates proliferation in culture systems, and that its breakdown drives tumorigenesis [314]. A recent mathematical study demonstrated that normal epithelial cells may suppress melanoma proliferation in co-culture [315]. This type of quantitative framework highlights the importance of capturing competition dynamics when modeling tumors in environments where malignant and healthy tissues coexist. Importantly, when such dynamics are incorporated into skin transport and pharmacokinetic models, they can guide the refinement of drug dosing strategies, improve predictions of diffusion depth across the stratum corneum, and help anticipate variability in therapeutic efficacy. By directly linking cellular competition to drug delivery modeling, these approaches bring mathematical oncology closer to informing the design of more precise and effective transdermal therapies for SC.

Figure 13 shows how interactions between normal and malignant skin cells, the regulation of contact inhibition, and the use of mathematical and computational approaches can predict outcomes in SC therapy, including optimizing drug dosing and diffusion and designing effective transdermal treatments. To the best of our knowledge, this integrated approach has not yet been fully explored in the literature.

To complete this overview, we will refer to the experimental models of melanoma discussed in the source [311], as well as the insightful review of computational models of melanoma found in [316], whose key takeaways include that even simple models can often be valuable, as noted in [317], especially in revealing hidden causal relationships. According to the authors, computational models focused on melanoma face four main challenges: tumor heterogeneity, melanoma subtypes, the need to balance simplicity and thoroughness in modeling, and the integration of melanoma data and evidence [317]. They concluded that a lack of interdisciplinary collaboration contributes to these challenges. Addressing them effectively requires close collaboration between experimental, clinical, and computational scientists. Such collaborations could bridge the gap between areas of knowledge and lead to promising advances in therapies within SC research [317].

Finally, to illustrate the preceding ideas, we will first highlight computational modeling tools used in the advanced stages of drug discovery, particularly in predicting drug delivery after initial in vitro testing. A notable example is an in vivo study in which dead cells in melanoma tumors provide abundant antigens for targeted delivery of ionizing radiation via a monoclonal antibody directed against melanin [318]. The computational model predicted effective delivery of a188-rhenium (188Re)-labeled monoclonal antibody (mAb) 6D2 (IgM) to metastatic melanoma tumors over a wide range of melanin concentrations [319]. In their sensitivity analyses, the researchers found that the dose reaching the tumor was reduced due to decreased diffusivity and increased lymphatic clearance from surrounding normal tissue [319]. This model was constructed using a system of partial differential equations that included plasma kinetics, transcapillary transport, interstitial diffusion, and lymphatic clearance. Michaelis–Menten kinetics was used to model mAb binding to tumor melanin; the reader is referred to the published article [300] for further information about this finding.

In addition, more advanced research has been conducted using a theranostic approach for imaging and treatment of melanoma using 203Pb/212Pb-labeled antibodies targeting melanin [320]. In these and other related studies [321], there is still potential for mathematical modeling in biodistribution studies [322] and in parameter estimation of ordinary differential equation models in pharmacokinetics [323,324,325], which can be useful for the selection of agents and procedures with the greatest potential for preclinical and clinical studies.

## 5. Concluding Remarks

This review synthesizes the multidisciplinary advances that position metallopharmaceuticals as credible options for SC therapy. By integrating the rational design of metallodrugs, where fine-tuning metal–ligand interactions, redox properties, and activation mechanisms can enhance selectivity and efficacy with chemical and physical penetration enhancers, topical delivery platforms, and nanocarrier strategies to stabilize labile complexes, together with light-activated modalities such as PDT, the field is addressing long-standing barriers related to limited skin permeation, chemical lability, and off-target toxicity. In parallel, mathematical and computational models ranging from diffusion-based descriptions to whole-skin biodistribution simulations provide quantitative guidance on dose, depth, and release, informing formulation refinement and the design of preclinical and clinical studies. The path forward is practical: standardize stability and safety assessments under in-use conditions; link model predictions to depth-resolved in-skin exposure and clinical endpoints; and deploy image-guided, depth-controlled, and patient-tailored regimens within local, effective, and safer SC treatments while minimizing systemic burden. Beyond delivery performance, a key next step for clinical translation is to incorporate subtype-specific biology and biomarker-informed stratification into study design and outcome interpretation. In practice, this means aligning metal-based mechanisms and formulation choices with clinically relevant features, particularly in non-melanoma skin cancers (SCC and BCC), which predominate in topical models, and leveraging established biomarker-guided decision axes in advanced melanoma (e.g., BRAF status and immune-related features) as a precedent for precision evaluation when local metal-based strategies advance.

As an additional forward-looking direction, polydopamine (PDA) enabled systems are emerging as pragmatic, biomimetic add-ons for skin-directed metallopharmaceutical platforms. PDA coatings provide a robust surface interface for local retention and device/nanocarrier functionalization, while their catechol/amine chemistry supports stable interactions with diverse payloads. In addition, PDA exhibits efficient near-infrared (NIR) photothermal conversion, enabling stimulus-responsive strategies in which light can spatially confine heating and support locally controlled activation in superficial lesions. Together, these features position PDA as a modular component to combine metal-centered activity with light-enabled control in topical or locoregional settings while remaining within a preclinical development landscape [326,327].

From a translational perspective, a relevant future direction is the integration of PDA-mediated photothermal priming with immunomodulatory components, including immune checkpoint blockade. Preclinical evidence supports that localized photothermal therapy can synergize with checkpoint inhibition in selected settings, providing a rationale to explore PDA-based photothermal systems as part of combination regimens. In skin cancer models, skin-directed PDA platforms incorporating metal elements, including transdermal microneedle-enabled approaches, illustrate feasibility for melanoma-focused proof-of-concept combinations. Importantly, these strategies should be framed as emerging rather than practice defining, and future work should prioritize standardized local tolerability reporting, depth-resolved exposure, and immunologic readouts to determine whether PDA–metal–checkpoint combinations can deliver clinically meaningful benefit beyond established standards [328,329,330].

It is also important to note that new metallopharmaceuticals and new administration strategies, followed by preclinical and clinical studies, are strongly required to define innovative and safe proposals for the systemic treatment of patients with disseminated cutaneous tumors.

## Figures and Tables

**Table 1:** Selected clinical examples of platinum-based agents and regimens in skin cancer and melanoma.

Agent (Metal + Route)	Skin Cancer Context	Clinical Setting/Phase	Key Clinical Takeaway	Notes for Wording (Avoid Overclaim)
Cisplatin (Pt(II); intratumoral) + electrochemotherapy (ECT)	Cutaneous tumor nodules/skin metastases (melanoma, SCC, and BCC)	Clinical experience/small clinical studies	Local responses reported; mainly local toxicity	Describe as skin-directed local therapy; avoid “approved for skin cancer” [129,130]
Cisplatin/epinephrine (adrenaline) injectable gel (intratumoral)	Cutaneous/soft-tissue melanoma metastases (skin confined)	Clinical study	Reported local activity; negligible systemic toxicity; local reactions manageable	Keep outcomes attributed to study setting/population “intralesional/intratumoral” [131]
Carboplatin (Pt; systemic) + paclitaxel (±sorafenib)	Metastatic melanoma	Phase III randomized trial	Used as chemotherapy backbone; trial assessed OS benefit of adding targeted agent	Evaluated in metastatic melanoma; not presented here as standard of care [132]
Carboplatin (Pt; systemic) + paclitaxel	Metastatic melanoma	Clinical regimen (multiple trials/series)	Activity reported but limited compared to modern standards	Frame as historical/selected use; avoid comparisons unless explicitly by cited trials [133,134]
Oxaliplatin (Pt; systemic)	Advanced/metastatic melanoma (exploratory)	Phase II (reported) trials	Explored in advanced melanoma; limited and not standard of care	Investigated in advanced melanoma; evidence is limited and not practice defining [135,136]

## Data Availability

No new data were created or analyzed in this study. Data sharing is not applicable to this article.
